# FGFR antagonists restore defective mandibular bone repair in a mouse model of osteochondrodysplasia

**DOI:** 10.1038/s41413-024-00385-x

**Published:** 2025-01-21

**Authors:** Anne Morice, Amélie de La Seiglière, Alexia Kany, Roman H. Khonsari, Morad Bensidhoum, Maria-Emilia Puig-Lombardi, Laurence Legeai Mallet

**Affiliations:** 1 Université de Paris Cité, Imagine Institute, Laboratory of Molecular and Physiopathological Bases of Osteochondrodysplasia, INSERM UMR 1163, Paris, France; 2 B3OA UMR CNRS 7052, Université Paris Cité, Paris, France; 3 Bioinformatics Core Platform, Imagine Institute, INSERM UMR1163 and Structure Fédérative de Recherche Necker, INSERM US24/CNRS UAR3633, Université Paris Cité, Paris, France

**Keywords:** Diseases, Physiology

## Abstract

Gain-of-function mutations in fibroblast growth factor receptor (FGFR) genes lead to chondrodysplasia and craniosynostoses. FGFR signaling has a key role in the formation and repair of the craniofacial skeleton. Here, we analyzed the impact of *Fgfr2*- and *Fgfr3*-activating mutations on mandibular bone formation and endochondral bone repair after non-stabilized mandibular fractures in mouse models of Crouzon syndrome (*Crz*) and hypochondroplasia (*Hch*). Bone mineralization of the calluses was abnormally high in *Crz* mice and abnormally low in *Hch* mice. The latter model presented pseudarthrosis and impaired chondrocyte differentiation. Spatial transcriptomic analyses of the *Hch* callus revealed abnormally low expression of *Col11, Col1a, Dmp1* genes in mature chondrocytes. We found that the expression of genes involved in autophagy and apoptosis (*Smad1, Comp, Birc2*) was significantly perturbed and that the *Dusp3*, *Dusp9*, *and Socs3* genes controlling the mitogen-activated protein kinase pathway were overexpressed. Lastly, we found that treatment with a tyrosine kinase inhibitor (BGJ398, infigratinib) or a C-type natriuretic peptide (BMN111, vosoritide) fully rescued the defective endochondral bone repair observed in *Hch* mice. Taken as a whole, our findings show that FGFR3 is a critical orchestrator of bone repair and provide a rationale for the development of potential treatments for patients with FGFR3-osteochondrodysplasia.

## Introduction

The fibroblast growth factor receptors (FGFRs) are a family of transmembrane receptor tyrosine kinases. Activation by the ligand fibroblast growth factor (FGF) leads to the intracellular activation of several pathways, including the rat sarcoma virus/mitogen-activated protein kinase (RAS/MAPK), phosphoinositide-3-kinase-protein kinase B (PI3K-AKT), phospholipase C gamma (PLCγ) and signal transducer and activator of transcription (STAT) pathways.^1^ The FGFRs and their ligands have a key role during the formation of the craniofacial skeleton.^2–12^ Cranial bone formation relies on membranous and endochondral ossifications. The formation of the calvarial bones and maxilla and cranial suture fusion depend on membranous ossification, while skull base formation depend on endochondral ossification.^13–15^ The formation of the mandibles requires both membranous and endochondral ossification: the horizontal branch of the mandible mainly relies on intramembranous ossification at Meckel’s cartilage (MC, a transient, antenatal cartilage that serves as a template), whereas the ascending branch, the condyle, and the symphysis are derived from endochondral ossification.^5,10,16^

In humans, activating *FGFR* mutations are responsible for a broad spectrum of osteochondrodysplastic diseases that lead to the abnormal development of the craniofacial and mandibular skeleton.^10,17^ Gain-of-function mutations in the *FGFR1*, *FGFR2* and *FGFR3* genes lead to craniosynostosis, i.e. the premature fusion of the skull base synchondrosis and one or more cranial sutures (including coronal sutures).^18^ The most frequent craniosynostoses is *FGFR2-*linked Crouzon syndrome (OMIM 123500), an autosomal dominant disorder that leads to craniofacial phenotypes of varying severity and that are characterized by brachycephaly, exorbitism, prognathism, and frontofacial retrusion.^19^ *FGFR3* mutations are also responsible for chondrodysplasia (such as hypochondroplasia (HCH) and achondroplasia (ACH)). HCH (OMIM 14600) is a mild-to-severe form^20,21^ of chondrodysplasia – an autosomal dominant disorder characterized by disproportionately short stature, short limbs, macrocephaly, frontal bossing, midfacial retrusion, and relative prognathism.^22,23^ With a view to characterizing the processes involved in mandible formation, researchers have developed a number of mouse models expressing the *Fgfr2* and *Fgfr3* gain-of-function mutations associated with craniosynostosis or chondrodysplasia. In a study of a mouse model of ACH (*Fgfr3*^*Y367C/+*^), our research group previously reported on abnormal mandibular shapes, mandibular hypoplasia, and defective chondrocyte proliferation and differentiation affecting MC and condylar cartilage.^10,24^ The impact of activating *Fgfr2* mutations in craniosynostoses has also been investigated in mouse models of Apert, Crouzon and Pfeiffer syndromes, with the observation of abnormal microarchitectures and mineralization of the developing mandible.^25^

The objective of the present study was to characterize the impact of heterozygous *Fgfr3* and *Fgfr2* gain-of function mutations on bone formation from antenatal period to adulthood and bone repair. We chose to study the recently developed *Hch* (*Fgfr3*^N534K/+^) mouse model,^26^ and the *Crz* mouse model (*Fgfr2c*^*C342Y/+*^) which shows coronal craniosynostosis with frontofacial retrusion, exorbitism, and mandibular anomalies.^3,25,27^ We observed that the mandible formation in *Hch* mouse model is impaired by a delay in MC resorption and by defective chondrocyte differentiation. In parallel, we studied the impact of *FGFR2-* and *FGFR3*- activating mutations during mandibular endochondral bone repair after non-stabilized mandibular fractures in *Crz* and *Hch* mouse models. The endochondral bone repair process involves the formation and then resorption of a transient cartilage; replacement of the latter with bone gives a soft callus that can bridge the bone defect.^28^ The impact of *Fgfr3* mutations on bone repair has been studied in various mouse models that showed severe consolidation delay and pseudarthrosis after non-stabilized fractures of the long bones.^29–31^ Despite the presence of these literature data, the impact of *Fgfr2* and *Fgfr3* activation on mandibular bone repair has not yet been characterized. To address the question, we analyzed mandibular bone fractures in both *Hch* and *Crz* mice. By studying bone repair after non-stabilized mandibular fractures in both mouse models, we demonstrated that the callus’ bone volume/total volume (BV/TV) ratio was higher after FGFR2 activation and lower after FGFR3 activation. Interestingly, anomalies in the cartilage callus were only observed in *Fgfr3* mouse model, which presented pseudarthrosis and severely abnormal consolidation. In order to characterize the impact of *Fgfr3* mutations on bone repair, we used whole-transcriptome analysis to measure the expression of specific markers involved in callus formation. Lastly, we performed pre-clinical studies using FGFR3 antagonists to investigate the effect of Fgfr3 modulation during bone repair in *Hch* mouse model. Interestingly, we found that two FGFR3 antagonists (BGJ398 and BMN111) can rescue bone repair in *Fgfr3*-related disorders. Taken as a whole, our results highlighted the various roles of FGFR2 and FGFR3 during endochondral bone repair and the positive action of FGFR3 antagonists in the context of fracture in FGFR3-related chondrodysplasia.

## Results

### The Fgfr3^N534K/+^ mutation affects mandible formation

**Fig. 1:**
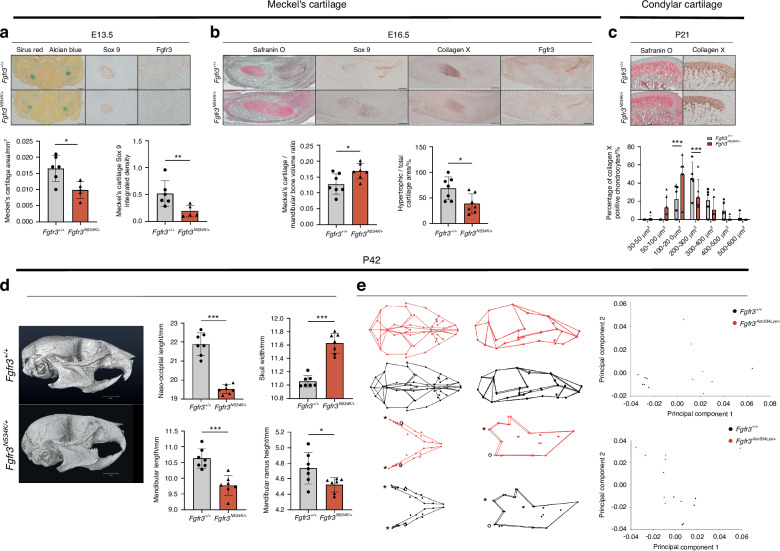
The *Fgfr3*^*N534K/+*^ mouse model of hypochondroplasia exhibits morphologic defects of the mandible. **a** Representative images of Meckel’s cartilage (MC) samples at E13.5 (Sirus Red/Alcian Blue staining). Scale bar: 200 µm. Sox 9 and FGFR3 immunostaining. Scale bar: 100 µm. Graphical representation of MC area and Sox9 staining. *Fgfr3*^*+/+*^ (*n* = 6) and *Fgfr3*^*N534K/+*^ mice (*n* = 5). **b** Representative images of *Fgfr3*^*+/+*^ and *Fgfr3*^*N534K/+*^ MC at E16.5 stained with Safranin O/Fast Green. Scale bar: 200 µm. Sox 9, collagen X and Fgfr3 immunostaining. Scale bar: 200 µm. Graphical representation of the MC area (Safranin O staining) and percentage ratio between hypertrophic area (collagen X) and total MC surface area (Safranin O). *Fgfr3*^*+/+*^ (*n* = 7) and *Fgfr3*^*N534K/+*^ (*n* = 7) mice. **c** Representative images of condylar cartilage at P21 from *Fgfr3*^*+/+*^ and *Fgfr3*^*N534K/+*^ mice (Safranin O/Fast Green) and collagen X immunostaining. Scale bar: 100 µm. Graphical representation of mean percentage of hypertrophic chondrocytes in different size categories (in µm^2^) inside the condylar cartilage of *Fgfr3*^*+/+*^ (*n* = 5) and *Fgfr3*^*N534K/+*^ mice (*n* = 6) (*n* > 50 cells for each genotype). **d** Representative µCT images of the skull bone at P42 from *Fgfr3*^*+/+*^ and *Fgfr3*^*N534K/+*^ mice (lateral orientation). Scale bar: 3 mm. Graphical representation of naso-occipital length, skull width, mandible length, mandibular ramus height (mm) from *Fgfr3*^*+/+*^ (*n* = 7) and *Fgfr3*^*N534K/+*^ mice (*n* = 7). **e** 3D representations of landmarks and associated wireframe variations in skull and mandibular shapes in P42 *Fgfr3*^*+/+*^ and *Fgfr3*^*N534K/+*^ mice after PC analysis. Shape comparison of skull and mandible (*: mandibular condyle; °: mandibular angle) (skull: Procrustes distance *d* = 0.109 4; *P* < 0.001; mandible: Procrustes distance *d* = 0.084 6; *P* < 0.001) in *Fgfr3*^*+/+*^ vs. *Fgfr3*^*N534K/+*^ mice, based on the scores for PC1 and PC2 (*n* = 7 mice for each genotype). *P < 0.05, **P < 0.01, ***P < 0.005

Mandible shape is influenced by extrinsic factors (linked to skull base changes) and intrinsic factors (linked to the mandibular cartilage’s formation and homeostasis).^10,32,33^ To assess the impact of *Fgfr3* gain-of-function mutations on mandibular bone formation, we took advantage of the recently developed *Hch* mouse model (*Fgfr3*^*N534K/+*^).^26^ We first determined whether or not the *Fgfr3*^*N534K/+*^ mutation impacted MC formation at E13.5 (Fig. 1a). The MC volume was significantly lower in *Hch* mice than in control littermates, with a relative reduction of 41.1% (*P* < 0.05; Fig. 1a). Immunostaining for Sox9 (a specific marker of early chondrocyte differentiation) revealed significantly lower expression of this protein in the MC in *Hch* mice, with a relative reduction of 71.9% vs. control littermates (*P* < 0.01; Fig. 1a). Interestingly, Fgfr3 was not expressed at E13.5 in MC of *Fgfr3*^*N534K/+*^ mice but was detected in control littermates. These data suggest that the onset of FGFR3 expression is delayed in the MC of *Fgfr3*^*N534K/+*^ mice and thus contributes to later MC formation. To determine the extent of chondrocyte proliferation, we studied the expression of proliferating cell nuclear antigen (PCNA). The staining intensity was lower in mutant mice than in control littermates (with a relative reduction of 62.2%) but the difference was not statistically significant (Fig. S1). At E16.5, we observed a significantly greater MC volume in *Fgfr3*^*N534K/+*^ mice (a relative increase of 43.7% vs. control littermates; *P* < 0.05) (Fig. 1b) and a significant lower proportion of area total cartilage with hypertrophic chondrocytes (marked by collagen type X) in the mutant mice (a relative reduction of 40.5%, vs. control littermates; *P* < 0.05; Fig. 1b). FGFR3 expression was more intense in *Fgfr3*^*N534K/+*^ mice than in control littermates (Fig. 1b). These data suggest that *Fgfr3*^*N534K/+*^ mutation delays MC resorption and impacts chondrocyte differentiation.

To further investigate the impact of the *Fgfr3*^*N534K/+*^ mutation on permanent cartilage, we analyzed the condylar cartilage during the postnatal period (P21). By analyzing collagen type X-positive chondrocytes, we observed a significantly higher proportion of smaller (100–200 µm^2^) hypertrophic chondrocytes in mutants (a relative reduction of 26.8% *vs.* control littermates; *P* < 0.005) and a significantly lower proportion of larger (200–300 µm^2^) chondrocytes (a relative increase of 20.6%; *P* < 0.005) (Fig. 1c). Hence, the *Fgfr3*^*N534K/+*^ mutation impacted chondrocyte differentiation in permanent cartilage, which might contribute to the postnatal limitation in mandibular growth.

We next used morphometric µCT to analyze mandibular shape and growth in adult mice (P42). We observed significantly lower values for mandible length (*P* < 0.005), ramus height (*P* < 0.05) and nasooccipital length (*P* < 0.005) and a significantly broader skull (*P* < 0.005) in mutant mice, when compared with control littermates (Fig. 1d). Next, we applied three-dimensional (3D) geometric analyses to further investigate differences in the craniofacial and mandibular shapes. *Fgfr3*^*N534K/+*^ mice displayed a more rounded skull, a shorter skull base, retrusion of the maxilla, and relative mandibular prognathism (Fig. 1e). As a result of the broader skull, the mandibular intercondylar and intergonial distances were greater in mutant mice than in controls (Fig. 1e). With regard to the maxillomandibular shapes, the first seven or first nine principal components (PCs) accounted for 90% of the total shape variation for the skull and for the mandible, respectively. A clear separation between mutants and controls was visible along PC1 as the result of significant shape differences (accounting for 39% and 23% of the total variance for the skull and the mandible, respectively; Fig. 1e; Procrustes distance for the skull: 0.109 4, *P* < 0.001; Procrustes distance for the mandible: 0.084 6, *P* < 0.001). Taken as a whole, our data indicate that (i) *Fgfr3* has a key role in MC, secondary cartilage and bone formation in the mandible and (ii) *Fgfr3* expression disturbs both membranous and endochondral ossification processes.

Previous studies of mandible formation in craniosynostoses have highlighted the role of *Fgfr2* expression in MC and mandibular periosteum, which induces MC enlargement, abnormal mandibular mineralization, and increased osteoclast activity.^5,11,25,27^ Overall, these data showed that FGFR2 and FGFR3 affect mandibular formation in different ways.

#### The *Fgfr2c*^*C342Y/+*^ mutation increases bone formation in the bone callus after non-stabilized mandibular fractures

**Fig. 2:**
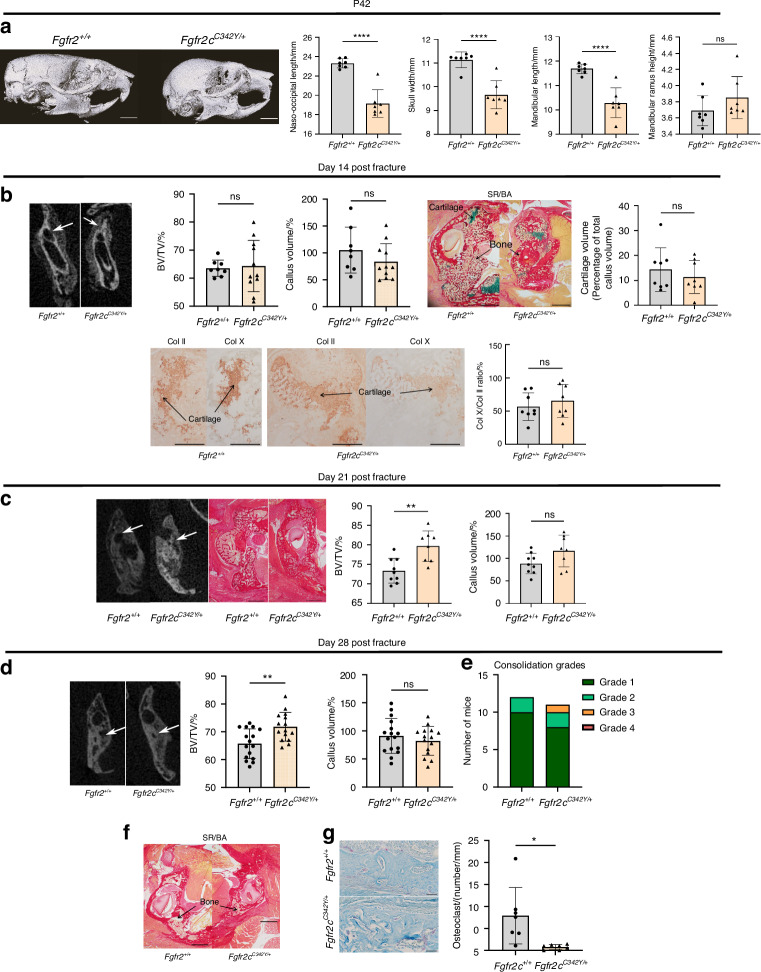
*Fgfr2c*^*C342Y/+*^ mutation increases bone formation in bone callus after non-stabilized mandibular fracture. **a** Representative µCT images of the skull bone in P42 *Fgfr2*^*+/+*^ and *Fgfr2c*^*C342Y/+*^ mice in lateral orientations. Scale bar: 3 mm. Graphical representation of the naso-occipital length, skull width, mandible length and mandibular ramus height (mm) in *Fgfr2*^*+/+*^ (*n* = 7) and *Fgfr2c*^*C342Y/+*^ (*n* = 7) mice. **b** Representative 3D coronal views at day 14 post-fracture of reconstructed µCT scans of mandibular fracture site (bone callus=white arrows) in *Fgfr2*^*+/+*^ and *Fgfr2c*^*C342Y/+*^ mice. Graphical BV/TV representation of the calluses from *Fgfr2*^*+/+*^ and *Fgfr2c*^*C342Y/+*^ mice. Graphical callus volumes representation from *Fgfr2*^*+/+*^ and *Fgfr2c*^*C342Y/+*^mice. Representative callus images of *Fgfr2*^*+/+*^ and *Fgfr2c*^*C342Y/+*^ (Sirus Red/Alcian Blue). Scale bar: 1 mm. Graphical representation of histomorphometric cartilage volumes on day 14 post-fracture (Alcian Blue/Sirus Red staining). Collagen II and X immunostaining of cartilage callus from *Fgfr2*^*+/+*^ and *Fgfr2c*^*C342Y/+*^. Scale bar: 500 µm. Graphical representation of percentage ratio between hypertrophic chondrocyte surface area (collagen X) and total chondrocyte surface area (collagen II). *Fgfr2*^*+/+*^ (*n* = 8) and *Fgfr2c*^*C342Y/+*^ (*n* = 8) mice. C: cartilage, B: bone callus. **c** Representative 3D coronal views at day 21 post-fracture of µCT scans of mandibular fracture site (bone callus=white arrows) in *Fgfr2*^*+/+*^ and *Fgfr2c*^*C342Y/+*^ mice. Representative images of the callus in *Fgfr2*^*+/+*^ and *Fgfr2c*^*C342Y/+*^ mice (Sirus Red/Alcian Blue). Scale bar: 1 mm. Graphical representation of BV/TV ratio in the calluses of *Fgfr2*^*+/+*^ and *Fgfr2c*^*C342Y/+*^ mice. Graphical representation of the callus volume in samples from *Fgfr2*^*+/+*^ and *Fgfr2c*^*C342Y/+*^mice. Representative images of callus in *Fgfr2*^*+/+*^ and *Fgfr2c*^*C342Y/+*^ mice (Sirus Red/Alcian Blue staining). Scale bar: 1 mm. *Fgfr2*^*+/+*^ (*n* = 9) and *Fgfr2c*^*C342Y/+*^ (*n* = 8). **d** Representative 3D coronal views at day 28 post-fracture of µCT scans of the mandibular fracture site in *Fgfr2*^*+/+*^ and *Fgfr2c*^*C342Y/+*^ mice (bone bridging = white arrows). Representative images of the callus in *Fgfr2*^*+/+*^ and *Fgfr2c*^*C342Y/+*^ mice (Sirus Red/Alcian Blue). Scale bar: 1 mm. Graphical representation of the BV/TV ratio in the calluses of *Fgfr2*^*+/+*^ and *Fgfr2c*^*C342Y/+*^ mice. Graphical representation of the callus volumes from *Fgfr2*^*+/+*^ and *Fgfr2c*^*C342Y/+*^ mice. **e** Graphical representation of consolidation grades: grade 1: total bone union; grade 2: bone union with minor bone defects; grade 3: partial bone union with major bone defects; grade 4: non-union. *Fgfr2*^*+/+*^ (*n* = 12) and *Fgfr2c*^*C342Y/+*^ mice (*n* = 11). **f** Representative images of mandibular calluses in *Fgfr2*^*+/+*^ (*n* = 17) and *Fgfr2c*^*C342Y/+*^ mice (*n* = 15) (Sirus Red/Alcian Blue staining), no residual cartilage or pseudarthrosis. Scale bar: 1 mm. B: bone callus. **g** Representative images of TRAP-labeled callus at day 28 post-fracture of *Fgfr2*^*+/+*^ and *Fgfr2c*^*C342Y/+*^ mice. Scale bar: 50 µm. Graphical representation of the osteoclast number per mm at the edge of the calluses (cortical bone). The data are quoted as the mean (SD); NS nonsignificant, *P < 0.05, **P < 0.01, ***P < 0.005, ****P < 10^−^^3^

We next examined the role of *Fgfr2* in bone repair. To this end, we created several series of non-stabilized vertical mandibular fractures of the ramus in adult (P42) *Fgfr2c*^*C342Y/+*^ mice and control littermates (Fig. S2A). We first noted significant morphometric differences between *Fgfr2*^*+/+*^ and *Fgfr2c*^*C342Y/+*^ mice with regard to the skull and mandibles, i.e. low values for naso-occipital length, skull width, and mandible length (*P* < 10^−^^3^), confirming the presence of craniofacial anomalies (Fig. 2a). Secondly, we studied bone repair and callus formation at various key points in the endochondral bone repair process (Fig. S2B). At day 14 post-fracture, morphometric analyses of CT data did not reveal any significant differences in the BV/TV ratio or callus volumes between *Fgfr2c*^*C342Y/+*^ mice and control littermates (Fig. 2b). Histological analyses on day 14 post-fracture did not evidence a significant impact of the *Fgfr2c*^*C342Y/+*^ mutation on callus cartilage volumes or the ratio of collagen X-positive to collagen II-positive areas (Fig. 2b). Terminal deoxynucleotidyl transferase dUTP nick end-labeling (TUNEL) experiments did not show any significant differences in the apoptosis rate for hypertrophic chondrocytes in cartilage calluses in *Fgfr2c*^*C342Y/+*^ mice vs. controls (Fig. S3). At day 21 post-fracture, we did not observe any persisting cartilage (i.e. no Sirus Red/Alcian Blue staining) in *Fgfr2c*^*C342Y/+*^ or control mice (Fig. 2c). No significant differences in callus volumes were observed between day 21 and day 28. In contrast, the callus BV/TV ratio was significantly higher in *Fgfr2c*^*C342Y/+*^ mice than in controls [10.6% higher on day 21 (Fig. 2c), *P* < 0.01; 7% higher on day 28, *P* < 0.01; Fig. 2d]. We next graded the quality of bone consolidation on day 28, using a 1-to-4 (best-to-worst) scale (Fig. 2e). Grade 1 or 2 bony bridging of the mandibular fractures was achieved in the control littermates (100%) and mutants (91%) (Fig. 2e). Non-union and persisting cartilage area were never observed in *Fgfr2c*^*C342Y/+*^ mice or control littermates (Fig. 2f). In agreement with these data, we found significantly lower numbers of osteoclasts at the callus boundary in *Fgfr2c*^*C342Y/+*^ mice, when compared with controls (a relative reduction of 90%, *P* < 10^−^^4^) (Fig. 2g). Overall, our results showed that the *Fgfr2c*^*C342Y/+*^ mutation did not have a significant impact on the initial stages of endochondral bone repair but did lead to greater bone formation and bone mineralization in the later stages of repair (i.e. during bone remodeling in the callus).

### Fgfr3^N534K/+^ mutation leads to defective mandibular bone repair and formation of pseudarthrosis

Experimental evidence shows clearly that *Fgfr3* gain-of function mutations impair bone formation and endochondral bone repair in long bones.^31,34^ In the present study, we used the same protocol as for *Fgfr2c*^*C342Y/+*^ mice to study bone repair and callus formation at various key points in the mandibular endochondral bone repair process in adult (P42) *Fgfr3*^*N534K/+*^ mice and control littermates (Fig. S2).

**Fig. 3:**
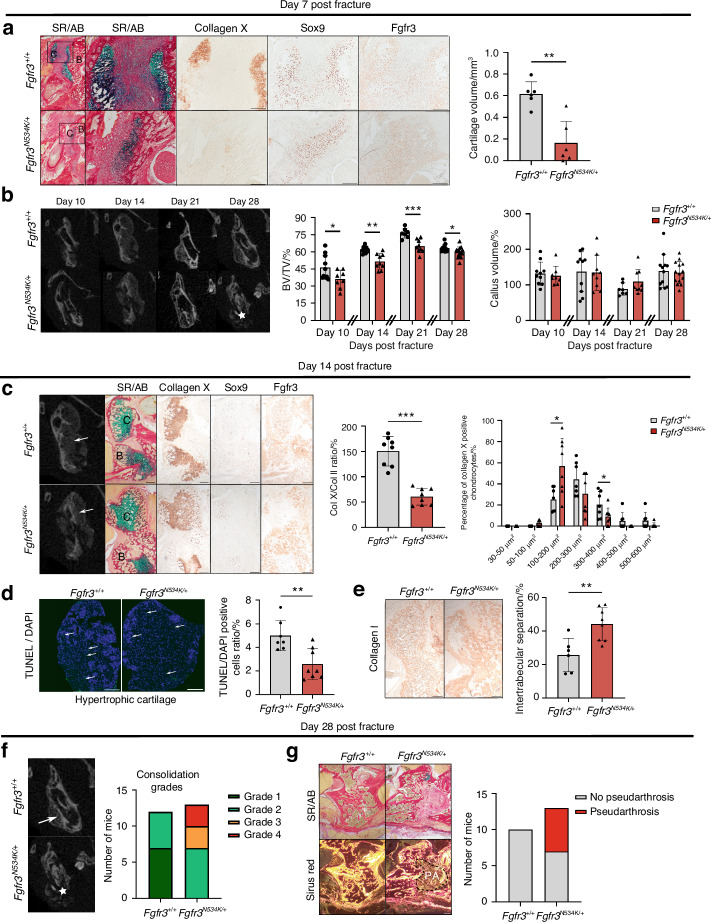
*The Fgfr3*^*N534K/+*^ *mutation is associated with defective mandibular bone repair and the formation of pseudarthrosis*. **a** Representative images of the calluses at day 7 post-fracture in *Fgfr3*^*+/+*^ and *Fgfr3*^*N534K/+*^ mice (Sirus Red/Alcian Blue staining). Scale bar: 200 µm. Magnification image of the cartilage callus (blue). Scale bar: 200 µm. Representative images of collagen X, Sox9, and Fgfr3 of cartilage callus from *Fgfr3*^*+/+*^ or *Fgfr3*^*N534K/+*^ mice. Scale bar: 200 µm. Graphical representation of histomorphometric cartilage volume (Alcian Blue/Sirus Red staining). *Fgfr3*^*+/+*^ *n* = 6, and *Fgfr3*^*N534K/+*^ *n* = 6. C: cartilage, B: bone callus. **b** Representative coronal views of 3D µCT scans of the mandibular fracture from day 10 to day 28 in *Fgfr3*^*+/+*^ and *Fgfr3*^*N534K/+*^ mice. Total bony bridging in *Fgfr3*^*+/+*^ and defective consolidation (*) in *Fgfr3*^*N534K/+*^ mice at day 28. Graphical representation of BV/TV in the calluses of *Fgfr3*^*+/+*^ and *Fgfr3*^*N534K/+*^ mice from day 10 to day 28 post-fracture. Graphical representation of callus volumes of *Fgfr3*^*+/+*^ and *Fgfr3*^*N534K/+*^ mice from day 10 to day 28 post-fracture. day 10, *n* = 10 *Fgfr3*^*+/+*^, *n* = 8 *Fgfr3*^*N534K/+*^; day 14, *n* = 10 *Fgfr3*^*+/+*^, *n* = 9 *Fgfr3*^*N534K/+*^; day 21, *n* = 7 *Fgfr3*^*+/+*^, *n* = 9 *Fgfr3*^*N534K/+*^; day 28, *n* = 12 *Fgfr3*^*+/+*^, *n* = 14 *Fgfr3*^*N534K/+*^. **c** Representative 3D coronal views of µCT scans at day 14 post-fracture of mandibular fracture site in *Fgfr3*^*+/+*^ and *Fgfr3*^*N534K/+*^ mice, bone callus (white arrows). Representative images of the callus cartilage in *Fgfr3*^*+/+*^ and *Fgfr3*^*N534K/+*^ mice (Sirus Red/Alcian Blue staining) and Collagen X, Sox9 and Fgfr3 immunostaining. Scale bar: 200 µm. Graphical representation of percentage ratio between hypertrophic chondrocyte surface area (collagen X staining) and total chondrocyte surface area (collagen II staining) in *Fgfr3*^*+/+*^ (*n* = 8) and *Fgfr3*^*N534K/+*^ (*n* = 8) mice. Graphical representation of the mean percentage of hypertrophic chondrocytes in different size categories (in µm^2^) in the callus of *Fgfr3*^*+/+*^ (*n* = 7) and *Fgfr3*^*N534K/+*^ (*n* = 8) mice (*n* > 100 cells for each genotype). C: cartilage, B: bone callus. **d** Representative images of TUNEL/DAPI staining (green fluorescence) at day 14 post-fracture of hypertrophic cartilage area (collagen-X-positive chondrocytes) of *Fgfr3*^*+/+*^ and *Fgfr3*^*N534K/+*^ mice. Graphical representation of the numbers of TUNEL- and DAPI-positive cells (%) in the calluses of *Fgfr3*^*+/+*^ (*n* = 7) and *Fgfr3*^*N534K/+*^ mice (*n* = 8). Scale bar: 100 µm. **e** Representative images of collagen I immunostaining at day 14 post-fracture of *Fgfr3*^*+/+*^ and *Fgfr3*^*N534K/+*^ mice. Scale bar: 200 µm. Quantification of the intertrabecular separation in bone callus from *Fgfr3*^*+/+*^ (*n* = 7) and *Fgfr3*^*N534K/+*^ (*n* = 8) mice. The data are quoted as the mean (SD); *P < 0.05, **P < 0.01, ***P < 0.005. **f** Representative 3D coronal views of µCT scans at day 28 post-fracture of *Fgfr3*^*+/+*^ and *Fgfr3*^*N534K/+*^ mice showing total bony bridging in *Fgfr3*^*+/+*^ mice and the absence of bone consolidation in *Fgfr3*^*N534K/+*^ mice (★). Graphical representation of consolidation grades: grade 1: total bone union; grade 2: bone union with minor bone defects; grade 3: partial bone union with major bone defects; grade 4: non-union. *Fgfr3*^*+/+*^ (*n* = 12), *Fgfr3*^*N534K/+*^ (*n* = 13). **g** Representative images (Sirus Red/Alcian Blue staining) of bone calluses at day 28 post-fracture of *Fgfr3*^*+/+*^ and *Fgfr3*^*N534K/+*^ mice, without polarization (above) and with polarization (below) showing fibrosis within the callus (i.e. pseudarthrosis). Scale bar: 100 µm. Pseudarthrosis in *Fgfr3*^*+/+*^ (*n* = 0/10) and *Fgfr3*^*N534K/+*^ (*n* = 6/13) mice

Histomorphometric analyses revealed significantly lower callus cartilage volumes on day 7 post-fracture in *Fgfr3*^*N534K/+*^ mice than in control littermates (a relative reduction of 88%, *P* < 0.01) (Fig. 3a). As expected, the expression levels of Sox9 and FGFR3 (Fig. 3a) and PCNA (*P* = 0.6) in cartilage callus were similar in *Fgfr3*^*N534K/+*^ mice vs. controls (Fig. S4a). Analysis of collagen type X expression revealed that maturation of the chondrocytes in *Fgfr3*^*N534K/+*^ cartilage was delayed, relative to controls (Fig. 3a). Morphometric analyses (based on CT scans of the callus) revealed that the BV/TV ratio was significantly reduced post-fracture in *Fgfr3*^*N534K/+*^ mice when compared with control littermates on day 10 (a relative reduction of 23%; *P* < 0.05), day 14 (a relative reduction of 14%; *P* < 0.01), day 21 (a relative reduction of 14.9%; *P* < 0.005), and day 28 (a relative reduction of 5.8%; *P* < 0.05) (Fig. 3b); There were no significant differences in the callus volume (Fig. 3b).

To gain further insights into the bone repair process, we analyzed the cartilage callus on day 14 post-fracture (Fig. 3c). The ratio of collagen X-positive/collagen II-positive areas was significantly lower in mutants than in control littermates (relative reduction: 62.8%; *P* < 0.005), demonstrating that *Fgfr3*^*N534K/+*^ mutation impairs hypertrophic chondrocyte differentiation (Fig. 3c). We noted that mutant mice had a significantly higher proportion of smaller chondrocytes (surface area: 100–200 µm^2^, *P* < 0.05; Fig. 3c), relative to the number of larger chondrocytes (surface area: 300–400 µm^2^); this finding indicated that the *Fgfr3*^*N534K/+*^ mutation impaired chondrocyte enlargement. The terminal regulation of chondrocyte differentiation involves many factors. In particular, we found that vascular endothelial growth factor (VEGF), a key protein in angiogenesis secreted by hypertrophic chondrocytes^35,36^ was more strongly expressed in mutants than in controls (Fig. S4b). In view of the impairment in chondrocyte maturation, we next sought to establish whether hypertrophic chondrocytes transdifferentiate into osteoblasts (forming the bone callus) or become apoptotic.^37^ To this end, we investigated apoptosis (using TUNEL assays) and observed a significantly lower proportion of apoptotic cells in collagen-X-positive areas of cartilage area (a relative reduction of 54.9%, compared with control littermates; *P* < 0.01; Fig. 3d). We next evaluated the expression of collagen I expression in the woven bone callus. Collagen I immunostaining revealed that the intertrabecular separation was significantly greater in mutants than in control littermates (a relative increase of 68.7%; *P* < 0.005; Fig. 3e). Lastly, we evaluated the quality of bone repair: total bony bridging of the mandibular fractures (grade 1 or 2) was achieved in all control littermate mice, whereas the absence of bone union (grade 4) was observed in 46% of the mutants and partial union (grade 2 or 3) was observed in 54% of the mutants (Fig. 3f). Using histomorphometric analyses, we gained further information on the tissues in grades 3 and 4 fracture repairs in *Fgfr3*^*N534K/+*^ mutants. Interestingly, we observed abnormal fibrosis and pseudarthrosis in these mutants (Fig. 3g). Taken as a whole, these findings demonstrate that endochondral mandibular bone repair is strongly impaired by *Fgfr3*^*N534K/+*^ mutation. In summary, callus formation and resorption are delayed in *Fgfr3*^*N534K/+*^ mice by defective chondrocyte differentiation and decreased apoptosis of hypertrophic chondrocyte, which lead to pseudoarthrosis.

### GeoMx spatial whole-transcriptome analyses of calluses in Fgfr3^N534K/+^ mice

**Fig. 4:**
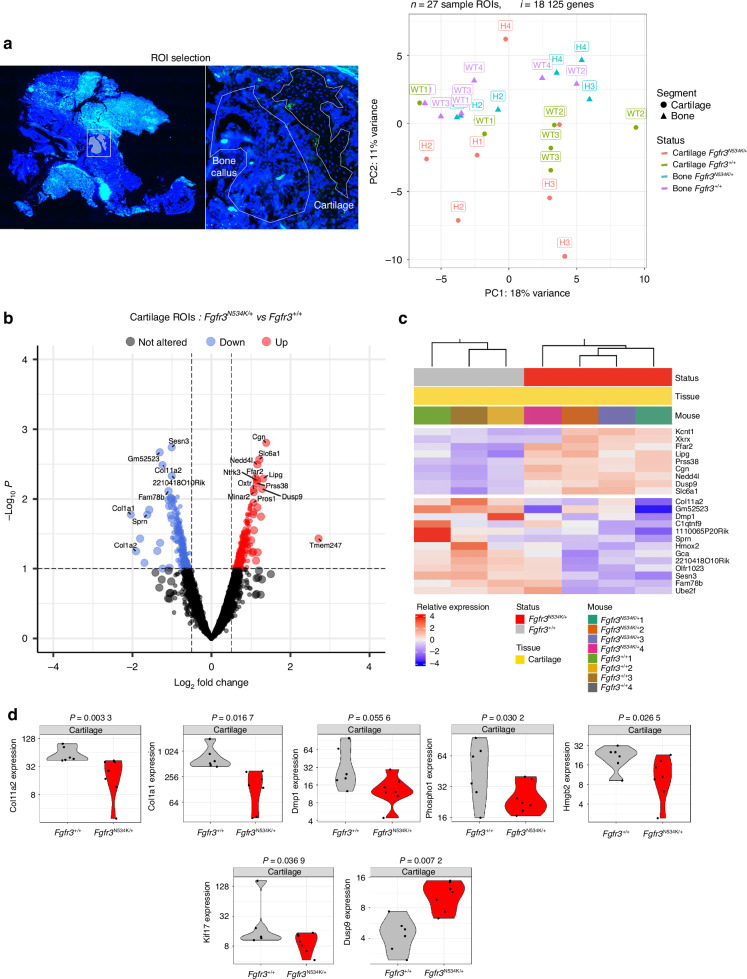
Spatial transcriptomic analysis of the cartilage in the calluses from *Fgfr3*^*N534K/+*^ and *Fgfr3*^*+/+*^ mice at day 14 post-fracture. **a** Slides were scanned for fluorescence, and ROIs were placed manually in areas of newly formed bone and cartilage in the callus. A PC analysis of transcriptome-wide normalized gene counts for bone and cartilage ROIs. All ROIs remaining after quality control filtering are shown, with each unique symbol and color pair indicating the genotype and the sample type (bone or cartilage), respectively. 27 ROIs and 18 125 genes were analyzed in four *Fgfr3*^*N534K/+*^ and four *Fgfr3*^*+/+*^ controls. **b**, **c** Volcano plot and heatmap representations of differentially expressed genes (*P* < 0.1) that were either upregulated (in red) or downregulated (in blue) in the cartilage of the calluses in *Fgfr3*^*N534K/+*^ mice, relative to *Fgfr3*^*+/+*^ mice. **d** Violin plots showing the expression levels (normalized read counts) of *Col11, Col1a1, Dmp1, Phospho1, Hmgb2, Kif17* and *Dusp9* in the cartilage of the calluses from *Fgfr3*^*N534K/+*^ mice (in red) and *Fgfr3*^*+/+*^ mice (in gray)

To further explore the molecular mechanisms affecting mandibular bone repair in the *Fgfr3*^*N534K/+*^ mouse model of hypochondroplasia and their control littermates, we conducted GeoMx spatial whole-transcriptome analyses of callus cartilage and newly formed bone on day 14 post-fracture. Twenty-seven regions of interest (ROIs) were selected for analysis (Fig. 4a).

In the callus cartilage, we assayed a total of 19 421 genes in the set of 27 ROIs; 8 211 genes were expressed in over 50% of the ROIs, and 18 125 were expressed in at least 10% of the ROIs. An unsupervised clustering analysis of the normalized read counts for these 18 125 genes revealed transcriptome-wide differences in expression between the samples (Fig. 4a). In order to examine specific expression changes between mutant and control ROIs, we performed differential expression analyses. Analysis of the cartilage ROIs in *Fgfr3*^*N534K/+*^ vs. *Fgfr3*^*+/+*^ mice identified 529 genes with significant (*P* < 0.1) changes in expression. Of these, 268 genes were upregulated [log_2_(fold-change) >0] in mutant samples relative to controls, and 261 were downregulated [log_2_(fold-change)<0]; (Fig. 4b, c). Gene set enrichment analysis of differentially expressed genes (based on the functional annotations in the Gene Ontology Biological Process (GO-BP) database) showed enrichments in specific pathways (Figs. S5a, S6a).

Specific cartilage markers (*Col2a1*, *Col10a*, *Sox9*, and *MAPK3*), *Pth* and *Bglap* were similarly expressed in both mutants and controls (Fig. S5b). In order to characterize the mechanisms underlying defective chondrocyte differentiation in the bone calluses in *Fgfr3*^*N534K/+*^ mice, we studied the expression of the *Hmgb2* gene coding for high mobility group box 2. The latter regulates chondrocyte differentiation and hypertrophy by mediating Runt-related transcription factor 2 expression and Wnt signaling.^38^ Our results highlighted the significant downregulation of *Hmgb2* in the cartilage callus of mutant mice (*P* = 0.02, Fig. 4d), relative to controls; this finding confirmed that the regulation of chondrocyte differentiation was impaired at the hypertrophic stage.

The gene coding for Indian Hedgehog (*Ihh*) is strongly expressed in cartilage. Indian Hedgehog stimulates chondrocyte proliferation and hypertrophy, prompts the transdifferentiation of chondrocytes into osteoblasts,^39^ and controls ciliogenesis^40–43^ Interestingly, we observed a low level of *Ihh* expression in mutant mice, although the difference with controls was not statistically significant (Fig. S5b). We hypothesize that weak *Ihh* expression disrupts chondrocytes.

We also explored the expression of genes involved in structural and functional aspects of primary cilia, such as the kinesin superfamily proteins (KIFs). These microtubule-based molecular motors convert the chemical energy of ATP hydrolysis into the mechanical force required to transport cargos along microtubules.^44,45^ We observed a significantly lower level of expression of *Kif17* (coding for kinesin-like protein 17, involved in the anterograde trafficking of ciliary proteins^46,47^) in the cartilage callus of *Fgfr3*^*N534K/+*^ mice, compared with control littermates (*P* = 0.03) (Fig. 4d). Low expression of *Kif17* is likely to disturb the primary cilia’s structure and function. Interestingly, we found that *Col11a2* (coding for collagen 11, essential for normal chondrocyte differentiation^48^) was strongly downregulated in mutants, compared with controls (*P* = 0.003) (Fig. 4d). We hypothesized that the lack of this collagen promotes the abnormal maturation of the hypertrophic chondrocytes. We next sought to determine whether or not the *Fgfr3*^*N534K/+*^ mutation impairs chondrocyte transdifferentiation. *Col1a1* (coding for an osteoblast marker^49^) was strongly and significantly downregulated in mutants, compared with control littermates (*P* = 0.01) (Fig. 4d). These data suggest that the transdifferentiation of the chondrocytes into osteoblasts does not occur during endochondral bone repair.

We also investigated the expression of genes involved in bone matrix mineralization and bone repair, such as *Dmp1* (coding for dentin matrix acidic phosphoprotein 1, a noncollagenous extracellular matrix protein from the small integrin-binding ligand, N-linked glycoprotein family)^50,51^ and *Phospho1* (coding for phosphatase, orphan 1, a skeletal tissue-specific phosphatase^52^). The levels of *Dmp1* and *Phospho 1* expression in the callus cartilage were significantly lower in *Fgfr3*^*N534K/+*^ mice than in control littermates (*P* = 0.05, *P* = 0.03, respectively) (Fig. 4d). Our data suggest that *Fgfr3*^*N534K/+*^ overactivation has a role in bone matrix formation during endochondral bone repair.

Furthermore, we studied the FGFR3’s downstream signaling pathways, such as the extracellular signal-regulated kinase (ERK), p38 MAPK, STAT, PI3K-AKT and protein kinase C pathways.^53^ In the *Fgfr3*^*N534K/+*^callus, we observed elevated levels of transcription of several subsets of MAPKs, although the differences were not statistically significant (Fig. S5b). Interestingly, *Dusp9* (coding for an ERK-specific phosphatase member of the dual-specificity (threonine/tyrosine) phosphatase superfamily) was significantly upregulated in the cartilage callus in *Fgfr3*^*N534K/+*^ mice (*P* = 0.007) (Fig. 4d); this observation confirmed the downregulation of excessive MAPK signaling pathway activity previously characterized in *Fgfr3* mouse models.^26,54^ Taken as a whole, these data suggest that the MAPKinase pathway is highly activated by the FGFR3-activating mutation in cartilage callus.

**Fig. 5:**
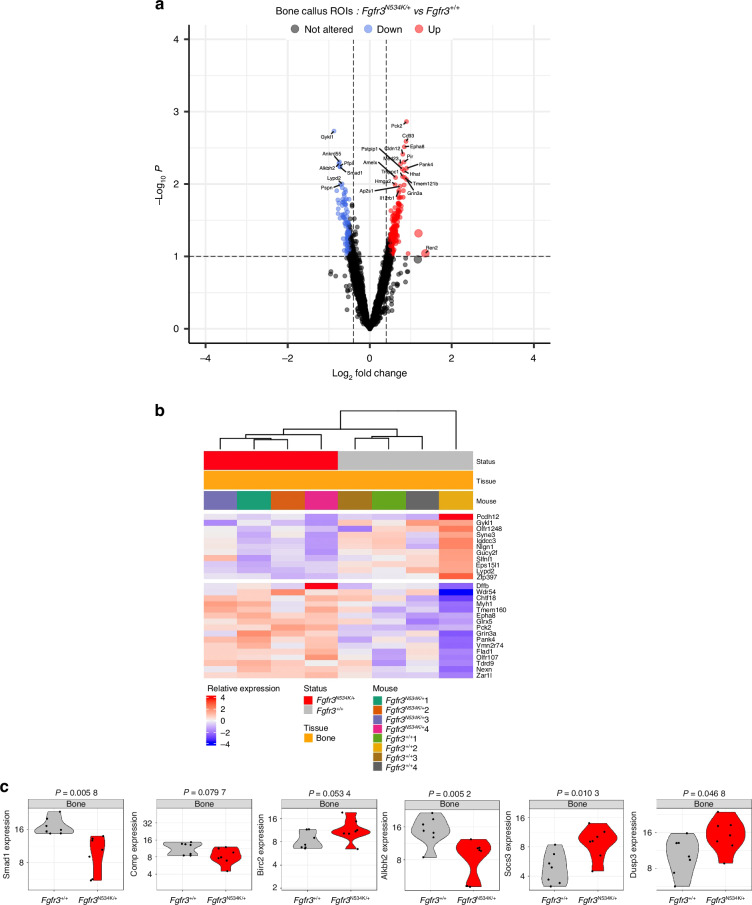
Spatial transcriptomic analysis of the newly formed bone in the calluses from *Fgfr3*^*N534K/+*^ and *Fgfr3*^*+/+*^ mice at day 14 post-fracture. **a** Volcano plot and **b** heatmap representation of the differentially expressed genes (*P* < 0.1) that were either upregulated (in red) or downregulated (in blue) in the bone calluses for *Fgfr3*^*N534K/+*^ mice, relative to *Fgfr3*^*+/+*^ mice. **c** Violin plots showing the expression levels (normalized read counts) of *Smad1, Comp, Birc2, Alkbh2, Dusp3* and *Socs3* in the newly formed bone of the calluses from *Fgfr3*^*N534K/+*^ mice (in red) and *Fgfr3*^*+/+*^ mice (in gray). The significance of the deregulation in *Fgfr3*^*N534K/+*^ mice is indicated by the *P*-values shown at the top

We next looked at how the FGFR3-activating mutation modulated gene expression in the bone callus. Comparative mutant vs. control analyses of the transcriptomic data from the newly formed bone ROIs identified 541 genes with significant (*P* < 0.1) changes in expression levels, relative to controls: 282 of these were upregulated [log_2_(fold-change) >0] and 259 were downregulated [log_2_(fold-change)<0]; (Fig. 5a, b). Gene set enrichment analysis of differentially expressed genes (based on functional annotation with the GO-BP database) revealed enrichment in several specific pathways (Figs. S5c, S6b). We did not find significant differences in the expression of the following osteoblast markers between *Fgfr3*^*N534K/+*^ mice and control littermates: *Col1a1*, *Osx (Sp7)*, *Runx2*, *Alpl* (coding for alkaline phosphatase), *Spp1* (coding for osteopontin), and *Tnfrs11a* (RANK, an osteoclast marker) (Fig. S5d).

Based on our finding that the *Fgfr3*^*N534K/+*^ mutation modified the balance between bone formation and resorption, we next examined biosynthetic and catabolic processes.^55,56^ In particular, we looked at specific genes involved in autophagy and apoptosis. Interestingly, we observed significant lower expression levels of *Smad1* in the bone calluses of *Fgfr3*^*N534K/+*^ mice, compared with control littermates (*P* = 0.005) (Fig. 5c). The bone morphogenetic protein (BMP)/Smad 1/5/8 pathway upregulates osteoblast differentiation and is activated by autophagy.^57^ Furthermore, we observed that the expression of *Comp* (coding for cartilage oligomeric matrix protein, a key regulator of the structural integrity of cartilage and an apoptosis suppressor) was significantly lower in mutants than in controls (*P* = 0.07). *Comp* suppresses apoptosis by inducing the inhibitor of apoptosis protein (IAP) family of survival proteins BIRC3, BIRC2, BIRC5, and XIAP.^58^ The expression of *Birc2* (*P* = 0.05) was greater in *Fgfr3*^*N534K/+*^ bone callus than in control littermates (Fig. 5c), indicating that the *Fgfr3*^*N534K/+*^ mutation disturbs the regulation of apoptosis during endochondral bone callus formation.

Given that apoptotic activity was seen to be abnormally elevated in the bone callus of *Fgfr3*^*N534K/+*^ mice, we next investigated markers of DNA repair. Human AlkB homolog 2 (ALKBH2) is a DNA repair enzyme that catalyzes the direct reversal of DNA methylation damage through oxidative demethylation and forms a complex with PCNA (especially during DNA replication^59^). Here, we found that the expression level of *Alkbh2* was significant lower in *Fgfr3*^*N534K/+*^ mice than in control littermates (*P* = 0.005) (Fig. 5c); this suggested that DNA repair was defective in the newly-forming bone callus. Lastly, we explored downregulators of the MAPK and STAT signaling pathways, which are activated downstream of FGFR3 activation and are responsible for defective chondrocyte differentiation and proliferation in *FGFR3*-linked chondrodysplasias.^1,54^ We found significantly higher expression levels of *Dusp3* (coding for a downregulator of MAPK pathways^60,61^) in *Fgfr3*^*N534K/+*^ mice compared to control littermates (*P* = 0.04) (Fig. 5c). We observed a significantly elevated expression level of *Socs3* (coding for suppressor of cytokine signaling-3, a downregulator of STAT3 signaling^62^ and MAPK^63^; *P* = 0.01) (Fig. 5c). Taken as a whole, our findings suggest that both MAPK and STAT pathways are over-activated in the bone callus in a mouse model of hypochondroplasia, and thus highlight the key role of these downstream signaling pathway in FGFR3-related disorders.

### BMN111 and BGJ398 protect against the onset of pseudarthrosis

**Fig. 6:**
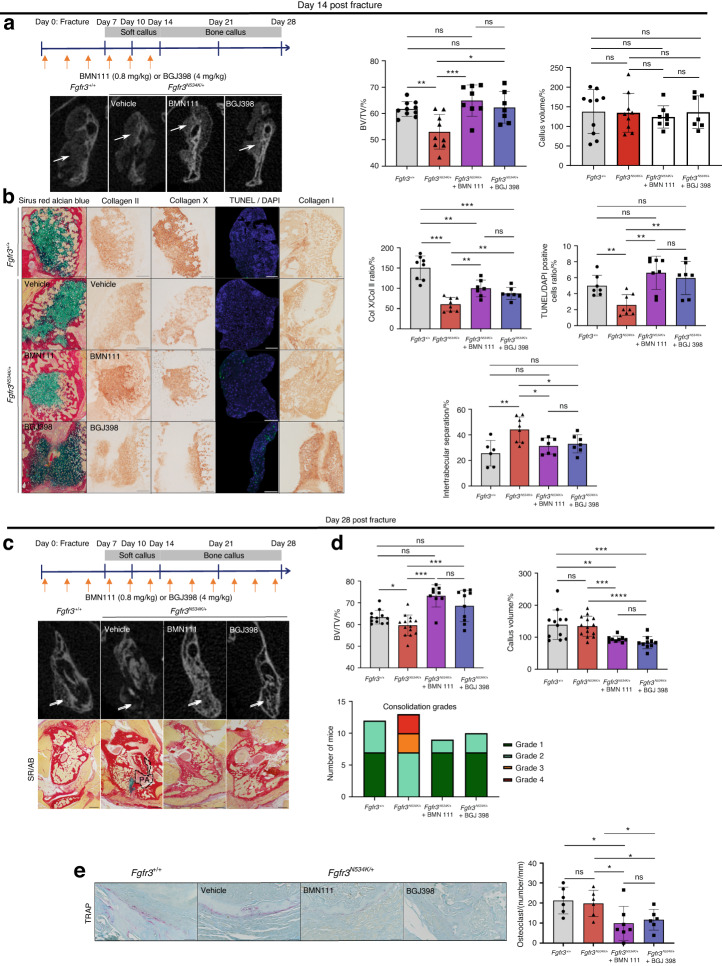
Defective bone repair with pseudarthrosis in *Fgfr3*^*N534K/+*^ mice is rescued by BMN111 and BGJ398. **a** Schematic representations of BMN111 and BGJ398 injections in *Fgfr3*^*N534K/+*^ mice, from day 0 to day 14 post-fracture. Representative 3D coronal views of µCT scans at day 14 post-fracture of *Fgfr3*^*+/+*^ and *Fgfr3*^*N534K/+*^ mice. Graphical representation of BV/TV in the calluses of *Fgfr3*^*+/+*^ and *Fgfr3*^*N534K/+*^ mice. Graphical representation of the callus volumes of *Fgfr3*^*+/+*^ and *Fgfr3*^*N534K/+*^ mice: *Fgfr3*^*+/+*^ + vehicle (*n* = 10), *Fgfr3*^*N534K/+*^ + vehicle (*n* = 9), *Fgfr3*^*N534K/+*^ + BMN111 (*n* = 8) and *Fgfr3*^*N534K/+*^ + BGJ398 (*n* = 7). **b** Representative images of the callus at day 14 post-fracture in *Fgfr3*^*+/+*^ and *Fgfr3*^*N534K/+*^ mice (Sirus Red/Alcian Blue staining). Representative images of collagen II, collagen X, TUNEL/DAPI and collagen I immunolabelling of the callus from *Fgfr3*^*+/+*^ and *Fgfr3*^*N534K/+*^mice. Scale bar: 200 µm. Graphical representation of collagen-X-positive chondrocyte/collagen-II-positive chondrocyte ratio: *Fgfr3*^*+/+*^ + vehicle (*n* = 8), *Fgfr3*^*N534K/+*^ + vehicle (*n* = 8) *Fgfr3*^*N534K/+*^ + BMN111 (*n* = 7), and *Fgfr3*^*N534K/+*^ + BGJ398 (*n* = 7). Graphical representation of TUNEL- and DAPI-positive cells in the hypertrophic chondrocyte area of the callus (in %): *Fgfr3*^*+/+*^ + vehicle (*n* = 7), *Fgfr3*^*N534K/+*^ + vehicle (*n* = 8), *Fgfr3*^*N534K/+*^ + BMN111 (*n* = 8), and *Fgfr3*^*N534K/+*^ + BGJ398 (*n* = 7). Graphical representation of intertrabecular area/total bone callus area ratio (collagen I staining): *Fgfr3*^*+/+*^+ vehicle (*n* = 6) *Fgfr3*^*N534K/+*^ + vehicle (*n* = 8), *Fgfr3*^*N534K/+*^ + BMN111 (*n* = 7) and *Fgfr3*^*N534K/+*^ + BGJ398 (*n* = 7). NS: non-significant, *P < 0.05, **P < 0.01, ***P < 0.005. **c** Schematic representation of BMN111 and BGJ398 injections in *Fgfr3*^*N534K/+*^ mutant mice from day 0 to day 28 post-fracture. Representative 3D coronal views of µCT scans at day 28 post-fracture showing the mandibular fracture site (white arrow). Representative images (Sirus Red/Alcian Blue staining) of the calluses at day 28 post-fracture from *Fgfr3*^*+/+*^ + vehicle, *Fgfr3*^*N534K/+*^ + vehicle, *Fgfr3*^*N534K/+*^ + BMN111, and *Fgfr3*^*N534K/+*^ + BGJ398, showing pseudarthrosis (PA) within the callus and persistent cartilage (blue) in *Fgfr3*^*N534K/+*^ mice, and continuous bone callus (total bridging) in *Fgfr3*^*+/+*^ + vehicle, *Fgfr3*^*N534K/+*^ + BMN111 and *Fgfr3*^*N534K/+*^ + BGJ398 mice. Scale bar: 1 mm. **d** Graphical representation of BV/TV of the calluses and the callus volume from *Fgfr3*^*+/+*^ and *Fgfr3*^*N534K/+*^ mice showing the benefit effect of the treatment. A graphical representation of consolidation grades after 28 days of treatment (grade 1: total bone union; grade 2: bone union with minor bone defects; grade 3: partial bone union with major bone defects; grade 4: non-union). *Fgfr3*^*+/+*^ + vehicle (*n* = 12), *Fgfr3*^*N534K/+*^ + vehicle (*n* = 14), *Fgfr3*^*N534K/+*^ + BMN111 (*n* = 9), and *Fgfr3*^*N534K/+*^ + BGJ398 (*n* = 10). **e** Representative images of TRAP*-*labeling at day 28 post-fracture in *Fgfr3*^*+/+*^ and *Fgfr3*^*N534K/+*^ callus. Scale bar: 100 µm. Graphical representation of the osteoclast count (osteoclasts per mm) at the edge of the calluses: *Fgfr3*^*+/+*^+ vehicle (*n* = 6), *Fgfr3*^*N534K/+*^ + vehicle (*n* = 6), *Fgfr3*^*N534K/+*^ + BMN111 (*n* = 7), and *Fgfr3*^*N534K/+*^ + BGJ398 (*n* = 6). NS: non-significant, *P < 0.05, **P < 0.01, ***P < 0.005, ****P < 0.000 5

BGJ398 (developed by Novartis Pharmaceuticals and also known as infigratinib) is a tyrosine kinase inhibitor that avoids the excessive phosphorylation of tyrosine kinase residues and corrects bone growth in our *Fgfr3*^*Y367C/+*^ mouse model.^8,10,54^ BMN111 (developed by BioMarin and also known as vosoritide) is a C-type natriuretic peptide (CNP) analog with a longer half-life than the native peptide. CNP inhibits the MAPK signaling activated by FGFR3. The positive effects of BMN111 on bone growth have been demonstrated in preclinical studies of *Fgfr3* mouse models^64,65^ and in clinical studies of children with achondroplasia.^66,67^ BMN111 was recently approved by the US Food and Drug Administration. Based on our previous findings that BMN111 treatment modulated the MAPK pathway^64^ and that BGJ398 treatment reduced the overactivation of FGFR3^54^ during development of the growth plate cartilage, we decided to examine the effects of these FGFR3 antagonists on endochondral bone repair. We again generated non-stabilized mandibular fractures in *Fgfr3*^*N534K/+*^ mice and treated the animals with either BMN111 (0.8 mg/kg), BGJ398 (4 mg/kg) or vehicle for 14 or 28 days post-fracture. At day 14 post-fracture, we morphometrically analyzed the callus (based on CT scans) in treated animals. There was no significant modification of the callus volume (Fig. 6a). The callus BV/TV values were significantly greater in drug-treated mutant mice than in vehicle-treated counterparts (29% greater for BMN111; *P* < 0.005; 20.8% greater for BGJ398; *P* < 0.05; Fig. 6a). There was no significant difference in the cartilage volume (Fig. S7). Interestingly, chondrocyte differentiation was significantly stimulated by both drugs, as shown by the significantly greater area ratio for collagen-X-positive chondrocytes vs. collagen-II positive chondrocytes (64.9% greater for BMN111; *P* < 0.01; 40.6% greater for BGJ398; *P* < 0.01; Fig. 6b). BMN111 treatment reduced the abnormally high expression of Sox9, VEGF and Fgfr3 protein immunostaining in *Fgfr3*^*N534K/+*^ mice, whereas the impact of BGJ398 on Sox9 expression was less marked (Fig. S4c). We next studied apoptosis: the proportion of TUNEL-positive cells in the hypertrophic chondrocyte area of the callus was significantly greater in both treated groups than in the control group (268% greater for BMN111; *P* < 0.01; 215% greater for BGJ398; *P* < 0.01). Our findings highlight the potential value of the two treatments for promoting apoptosis in the callus (Fig. 6b). We also studied the bone part of the callus: collagen I immunostaining revealed that the intertrabecular separation was significantly smaller in the drug-treated groups than in the control group (26.5% smaller for BMN111; *P* < 0.05; 28.7% smaller for BGJ398; *P* < 0.05). Both treatments promoted bone formation in the calluses by day 14 (Fig. 6b). We next analyzed the fractured mandibles at the end of the bone repair process (day 28 post-fracture) (Fig. 6c). The BV/TV ratio was significantly higher in mice treated with BMN111 (24% higher; *P* < 0.005) or BGJ398 (17.8% higher; *P* < 0.01) (Fig. 6d). Interestingly, callus volumes were significantly lower in both groups of treated mice (30.9% lower for BMN111; *P* < 0.005; 38.6% lower for BGJ398; *P* < 0.000 1; Fig. 6d). Lastly, an analysis of the μCT scans evidenced complete bony bridging in both groups and an absence of pseudarthrosis in all treated *Hch* mice, confirmed by histological analysis (Fig. 6c). We next assessed osteoclast activity at the boundary of the calluses, using tartrate-resistant acid phosphatase (TRAP) staining. The osteoclast count was significantly lower in both groups of treated mutants (66.8% lower for BMN111; *P* < 0.05; 43.8% lower for BGJ398; *P* < 0.05) (Fig. 6e), showing that bone resorption was stimulated by BMN111 and BGJ398. Our findings show that bone callus remodeling by day 28 post-fracture is accelerated by BGJ398 and BMN111 (Fig. 6d). Overall, these data highlight the potential for clinical application of FGFR3 antagonists in bone callus remodeling after non-stabilized mandibular fracture.

## Discussion

The specific roles of *Fgfr2* and *Fgfr3* during bone formation and repair had not previously been studied in depth. We first reported that the *Fgfr3*^*N534K/+*^ mouse model of hypochondroplasia presented particular craniofacial and mandibular phenotypic features.^26^ In the early stages of MC formation (E13.5),^68^ the *Fgfr3*^*N534K/+*^ mutation leads to a delay in the onset of chondrocyte proliferation and to defective chondrocyte differentiation at E16.5. At P14, we observed significant size and shape differences in *Fgfr3*^*N534K/+*^ mutant mice: retrusion of the maxilla, relative mandibular prognathism, and a shorter mandible.^26^ From P14^26^ to P42 (the present report), the craniofacial and mandibular phenotypes became accentuated with age. Endochondral ossification in the condylar region allows ramus elongation. Importantly, we observed an impairment in chondrocyte maturation in the condylar cartilages during mandibular growth (at P21), which shows that the *Fgfr3*^*N534K/+*^ mutation is associated with major differences in the post-natal endochondral ossification of the mandible. These results show clearly how the severity of the mandibular phenotype increases with age in *Hch* mice.

The impact of *Fgfr2*-activating mutations on MC homeostasis has already been studied in *Fgfr2* craniosynostosis mouse models of Apert, Crouzon and Pfeiffer syndromes.^25,27^ Low proliferation in MC (E15.5) and in the forming mandible (E17.5) has been observed in *Fgfr2c*^*C342Y/+*^ embryos.^27^ In contrast to the situation for the *Fgfr3*^*N534K/+*^ *Hch* mouse model, we did not observe a significant impact of the *Fgfr2c*^*C342Y/+*^ mutation on chondrocyte differentiation in MC in our Crouzon embryos at E16.5 (data not shown).

Next, we investigated the impact of *Fgfr2*- and *Fgfr3*- activating mutations on endochondral bone repair after non-stabilized fractures of the mandible in *Hch* and Crouzon mouse models and controls. The chondrocytes invade the callus concomitantly to vascular invasion, from day 2 post-fracture onwards.^69–71^ In *Hch* mice, endochondral bone repair was defective and was characterized by poor chondrocyte differentiation and a low apoptosis rate in the mandibular callus. These changes resulted in non-bone union with pseudarthrosis in nearly half of the *Fgfr3*^*N534K/+*^ mice and consolidation was delayed in all cases. These data are in line with the reported presence of pseudarthrosis after non-stabilized tibial fractures in *Prx1*^*Cre*^*;Fgfr3*^*Y367C/+*^ mutants and confirm the key role of *Fgfr3* in bone repair.^31^

Interestingly, we observed significant microarchitectural alterations in bone calluses from day 10 to day 28 post-fracture, without excessive bone resorption. The defective bone formation during mandibular bone repair observed in *Hch* mice, corroborates with our previous findings in the same *Hch* mouse model concerning the long bones.^26^ We observed biomechanical anomalies, characterized by increased bone fragility with microarchitectural alterations of the long bones, highlighting that *Fgfr3* activating mutations affects both bone formation, repair and remodeling of bones of different embryological origin.^26^ Defective bone callus mineralization has also been observed after distraction osteogenesis of the long bones in ovariectomized mice bearing the *Fgfr3*^*G380R/+*^ mutation (a model of achondroplasia).^72^ All these data highlight the key role of *Fgfr3* in bone repair after non-stabilized fractures and distraction osteogenesis.

Spatial whole-transcriptome analyses of the callus highlighted differences in the expression of some genes. This novel approach enables us to characterize the role of key genes during the endochondral bone process, with a focus on bone and cartilage. Interestingly, expression of *Col11a2* (which is essential for normal chondrocyte differentiation^48^) was downregulated in the cartilage callus in *Fgfr3*^*N534K/+*^ mice. This was also the case for IHH, the product of which is a key regulator of chondrocyte proliferation and differentiation and a promotor of chondrocyte-to-osteoblast transdifferentiation.^39^ Low levels of Ihh were observed in chondrocytes isolated from another Fgfr3 mouse model of achondroplasia (*Fgfr3*^*Y367C/+*^).^43^ Furthermore, our analyses revealed downregulation of specific osteoblast markers that are usually expressed by hypertrophic chondrocytes with an osteoblast-like cell fate during transdifferentiation (i.e. *Col1a1*, *Dmp1*, and *Phospho1*) and several markers involved in bone matrix mineralization (including *Dmp1*^50–52,73^). Our results indicate that transdifferentiation is not favored in bone matrix mineralization.

The regulation of chondrocyte differentiation is impaired in *Hch* mice, as revealed by the significantly downregulation of *Hmgb2* (coding for high mobility group box 2, a regulator of chondrocyte hypertrophy^38^) in *Fgfr3*^*N534K/+*^ mice. The role of *Hmgb2* during endochondral bone repair has not been studied previously. Our present data suggest that *Hmgb2* downregulation modulates the defective hypertrophic chondrocyte differentiation due to FGFR3 activation. Interestingly, ciliary gene mutations are responsible for many skeletal ciliopathies, and the expression of ciliary components is also modified in *Hch* mice; we observed downregulation of *Kif17* (coding for kinesin-like protein 17, involved in the anterograde trafficking of ciliary proteins).^46,47^ These findings are in agreement with previous reports of defective ciliogenesis in the *Fgfr3*^*Y367C/+*^ mouse model^43^ and further suggest that *Fgfr3*^*N534K/+*^ mutation leads to defective intraciliary protein trafficking, which might in turn disturb chondrocyte organization in the callus cartilage. In parallel, our analyses of the newly formed woven bone in the calluses did not evidence any differences in levels of the osteoblast markers *Col1a1*, *Osx (Sp7)*, *Runx2*, *Alpl* (coding for alkaline phosphatase), *Spp1* (coding for osteopontin) and the osteoclast marker *Tnfrs11a* (coding for RANK) after the activation of FGFR3. Given that bone homeostasis depends on the balance between bone formation and bone resorption and involves both synthesis and catabolic processes, we investigated the impact of the *Fgfr3*^*N534K/+*^ mutation on autophagy and apoptosis.^55,56^ It is known that FGF/FGFR signaling has a role in the regulation of autophagy^56^ and that FGFR3 activation inhibits autophagic processes during skeletal bone formation and growth.^74^ Defective autophagy leads to endoplasmic reticulum stress, which in turns downregulates the BMP/Smad1/5/8 pathway and inhibits osteoblast differentiation.^57^ Here, we found that *Smad1* was significantly downregulated in the bone calluses of *Fgfr3*^*N534K/+*^ mice. We speculate that *Smad 1* downregulation is due to the defect in autophagy linked to *Fgfr3* overexpression, which in turn might inhibit osteoblast differentiation. Next, we found that apoptosis regulation was perturbated in the bone calluses of *Fgfr3*^*N534K/+*^ mice, as shown by the downregulation of *Comp* (coding for cartilage oligomeric matrix protein, a suppressor of apoptosis^58^ expressed in both chondrocytes and osteoblasts in developing and mature tissue^75,76^). Our results corroborate a report on another mouse model of pseudo-achondroplasia (the homozygous CompT585M knock-in model) characterized by disorganization of the growth plates, dysregulated apoptosis, and short limb dwarfism.^77^ Lastly, we observed impaired DNA repair in *Fgfr3*^*N534K/+*^ mice, as revealed by the downregulation of *Alkbh2* (coding for a DNA repair enzyme that catalyzes the direct reversal of DNA methylation damage through oxidative demethylation).^59^ Overall, these results indicate that the *Fgfr3*^*N534K/+*^ mutation disturbs autophagy and apoptosis during bone callus formation. Furthermore, we studied the gene expression of members of the dual-specificity (threonine/tyrosine) phosphatase superfamily (Dusp),^60,61^ which downregulate the MAPK and STAT signaling pathways downstream of Fgfr3.^54,78^ Interestingly, *Dusp9* and *Dusp3* (coding for downregulators of the MAPK pathway) were upregulated respectively in the cartilage and in the bone callus of *Fgfr3*^*N534K/+*^ mice. We also observed significantly higher expression levels of *Socs3* (coding for suppressor of cytokine signaling-3, a downregulator of STAT3 signaling^62^ and MAPK signaling).^63^ Overall, our data confirm that the MAPK pathway is a key downstream signaling pathway controlling chondrocyte differentiation and bone formation in the context of constitutive *Fgfr3* activation. Our data also suggest that impaired bone callus formation is due to the effects of *Fgfr3* overexpression on chondrocyte and osteoblast differentiation via the MAPK pathway and via downregulation of the BMP/Smad pathway in response to defective autophagy.

Our analyses of bone repair in Crouzon *Fgfr2c*^*C342Y/+*^ mice were also very informative. We did not observe a significant impact of *Fgfr2c*^*C342Y/+*^ mutation on early-stage endochondral mandibular bone repair but we did measure greater bone mineralization at later stages of bone consolidation. These findings are in line with the known impact of the *Fgfr2c*^*C342Y/+*^ mutation, i.e. excessive bone mineralization during coronal suture formation, leading to premature suture fusion.^79^ In contrast to our results in the mouse model of hypochondroplasia, we did not observe a marked consolidation delay or pseudarthrosis in *Fgfr2c*^*C342Y/+*^ mutants. The various impacts of the mutations on callus formation might be explained by differences in the expression of *Fgfr2* and *Fgfr3* during bone formation and repair. *Fgfr2* is expressed from the early stages of endochondral bone repair (day 1 post-fracture) onwards by periosteal cells, chondrocytes and osteoblasts, whereas *Fgfr3* is expressed from day 4 post-fracture by mesenchymal cells, prehypertrophic and hypertrophic chondrocytes, osteoblasts, and periosteal cells.^80–82^ Unlike *Fgfr3* expression (which decreases from day 9 post-fracture onwards), *Fgfr2* expression remains high.^80^ Overall, our results demonstrate that *Fgfr3* is a key regulator of cartilage formation and differentiation during endochondral bone repair, and that *Fgfr2* has a more limited role in callus cartilage and preferentially acts on osteoblast differentiation at later stages (i.e. during bone remodeling).

To determine whether FGFR3 antagonists could rescue the defective bone repair observed in vivo in our *Hch* mouse model, we evaluated the action of the drugs BGJ398 and BMN111 in mutant mice. Interestingly, both drug treatments resulted in normal consolidation. Chondrocyte differentiation and apoptosis were improved in cartilage callus, and microarchitectural alterations in the bone callus were rescued by both treatments. Interestingly, BMN111 mostly rescued chondrocyte differentiation, whereas BGJ398 mostly stimulated osteoblast differentiation and maturation. Both drug treatments accelerated bone repair and changed the bone formation/bone resorption balance so that bone apposition could restore the mandibular cortical bone.

In conclusion, a *Fgfr3* gain-of-function mutation was associated with major impairments in endochondral bone formation and mandible repair and promoted the formation of pseudarthrosis. Our findings highlight a major role of Fgfr3 in the regulation of chondrocyte and osteoblast differentiation during bone repair. Moreover, our results further support the concept whereby FGFR3 antagonists can rescue impaired bone callus formation in the context of *Fgfr3* gain-of-function mutations. These data might open up promising therapeutic perspectives for the restoration of defective bone repair in people with FGFR3-related osteochondrodysplasia, in cases of traumatic bone fractures, and in patients having undergone craniomaxillofacial and mandibular osteotomies. Further research is needed to determine whether or not the modulation of FGFR3 activity by cognate antagonists accelerates bone repair under normal conditions (i.e. in the absence of *FGFR* mutations). In the future, the pharmacological modulation of FGFR3 activity might provide treatment options for patients with severe, persistent consolidation defects.

## Material and methods

### Mouse models and study approval

All the experiments were conducted in two mouse models of osteochondrodysplasia: a *Fgfr2c*^*C342Y/+*^ mouse model of Crouzon craniosynostosis, and a *Fgfr3*^*N534K/+*^ mouse model of hypochondroplasia. The *Fgfr2c*^*C342Y/+*^ mouse model was generated by Eswarakumar et al. and mimics some of the clinical features of human coronal craniosynosotosis.^3^ All the *Fgfr2c*^*C342Y/+*^ mice and their controls had a CD1 background. The *Fgfr3*^*N534K/+*^ (*Fgfr3*^*Asn534Lys/+*^*)* mouse model mimics some of the clinical features of human hypochondroplasia;^26^ wild-type littermates were used as controls. The *Fgfr3*^*Asn534Lys/+*^ mutant mice expressed the Asn534Lys mutation in exon 12 of *Fgfr3* (corresponding to Asn540Lys in human) and the loxP-flanked neo/STOP cassette. The *Fgfr3*^*Asn534Lys/+*^ mice and controls had a C57BL/6 background.

Mice were genotyped using PCRs of total DNA extracted from a sample of external ear tissue. Animal procedures were approved by the French Ministry of Research and the Animal Care and Use Committee at Université Paris Cité (APAFIS 26995) and were conducted in compliance with ethical principles at the LEAT Facility (Imagine Institute, Paris, France).

In all analyses, wild-type littermates were used as controls. Both males and females were used in experiments because no significant sex differences have been observed for the biological processes studied herein.

### Non-stabilized mandibular fractures

Six-week-old female and male mutant and control mice were anesthetized with isoflurane (induction: 4%; maintenance: 2.5%) and received a preoperative subcutaneous injection of buprenorphine (0.1 mg/kg). Non-stabilized mandibular fractures were created via a submandibular approach. A cutaneous incision was made along the inferior (basilar) border of the right mandible. The masseter muscle was elevated into the subperiosteal plane along the ascending branch of the mandible, using a periosteal elevator.

A vertical, complete fracture of the ascending branch was created from the basilar border to the coronoid notch (between the condylar and the coronoid processes), using a piezotome, saline irrigation, and scissors (Fig. 2a). The skin incision was closed with silk suture. Sagittal-plane X-rays of the head were performed immediately after the operation, in order to check the position of the mandibular fracture.

Mice received subcutaneous injections of buprenorphine (0.1 mg/kg) 4 h and 1, 2 and 3 days after surgery. Throughout the postoperative period, mice received soft chow. Food intake, body weight and activity were monitored every day for the first week and then every other day or as required. In the event of excessive mandibular incisor growth in mutants (due to shortened skull base, maxillary retrusion and prognathism) and to avoid feeding difficulties, the teeth were cut as necessary before and/or after surgery. The entire skull and mandibles were collected at 7, 10, 14, 21 and 28 days post-fracture.

### Microradiography

X-ray imaging was performed immediately after the fracture and post-mortem, using a Faxitron UltraFocus digital radiography system (Faxitron Bioptics, Tucson, AZ, USA). Lateral images of the head and mandibles were taken at a 1x magnification.

### CT images

Micro-CT imaging of the skull and mandibles was performed with a Skyscan 1172 system (Bruker MicroCT NV, Kontich, Belgium). For morphological analyses, the skull and mandibles of mutants and controls (age: P42) were fixed in 70% ethanol. For analyses of bone repair, the samples consisted of mandibles of mutants and controls (euthanized on day 10, 14, 21 or 28-post-fracture) fixed in paraformaldehyde (4%) for 24 h, washed in 1XPBS, and scanned immediately.

The scan settings were as follows: 80 kV, 100 μA, a pixel resolution of 23 μm, rotation steps of 0.5°, and a 0.5 mm aluminum filter. Three-dimensional reconstructions were generated using Nrecon software (Bruker MicroCT NV). The bone density threshold was adjusted for each CT scan, with no smoothing.

For morphometric analyses of bone repair, the ROI (the callus) was selected manually using CTAn software (Bruker MicroCT NV). The BV/TV ratio for each callus was automatically calculated, using CTAn software. To compare callus volumes in controls vs. mutants presenting significantly different mandible sizes, the entire contralateral unfractured ascending branch of the mandible was contoured manually from the retromolar area. For normalization, the ratio between the TV of the callus and the TV of the contralateral ascending branch was calculated.

For 3D geometric morphometric analyses, the reconstruction surface mesh for each specimen was obtained using Avizo® software (ThermoFisher Scientific, Waltham, MA, USA) and then used for landmark positioning of the skull (Fig. S8a and Supplemental File 1) and mandibles (Fig. S8b and Supplemental File 1). Landmark 3D coordinates were analyzed using geometric morphometric methods, including standardization for position, scale, and orientation through Procrustes superimposition.^83^ Measurement errors were assessed with a Procrustes analysis of variance; the absence of significant differences in landmark positioning enabled valid, comparative analyses of shape variation from one specimen to another. After Procrustes superimposition, the resulting 3D Procrustes shape coordinates were evaluated in a PC analysis. Wireframes were used to visualize the shape differences corresponding to the skull and associated with specific scores for PCs. Centroid size (CS, the square root of the sum of squared distances between the landmarks and their centroid) was used as a proxy for size.^84^ The influence of size on shape (i.e. allometry) was tested with multiple multivariate regression. Procrustes distance (d) separating groups in the morphospace were computed, and statistical significance was assessed in permutation tests (10 000 permutation rounds). A significant value of d was interpreted as a significant shape difference between the two groups compared. The geometric morphometric analyses were performed with MorphoJ software (version 1.06).^85^

To evaluate the degree of bone consolidation, we established the following scale; grade 1: total bone union; grade 2: bone union with minor bone defects; grade 3: partial bone union with major bone defects: grade 4: non-union.

### Histology

All samples were collected in 4% paraformaldehyde, fully decalcified in 0.8 mol/L EDTA (pH 8.0) and embedded in paraffin. Six µm serial sections were prepared. All slices were deparaffinized in Neo-clear solution (Merck Millipore, Darmstadt, Germany) and then rehydrated prior to standard immunostaining. Sections were stained with hematoxylin/eosin, Safranin-O/Fast-Green, or Sirus Red/Alcian Blue, using standard protocols.

Sections were stained with Safranin O for evaluation of the MC volume and with Sirus Red/Alcian blue for evaluation of the cartilage and bone volumes of the calluses. At least seven equidistant sections at 120 µm intervals throughout the callus were analyzed for each callus. The cartilage, bone TVs in the ROIs were estimated using Cavalieri’s principle, as described by Abou-Khalil et al. ^86^.

Selected adjacent slides were stained using standard immunohistochemical protocols and antibodies against collagen I (1:100, 20151-1, Novotec, Lyon, France), collagen II (1:500, 20251, Novotec, Lyon, France), collagen X (1:100, 1-CO097-05, Quartett, Berlin, Germany), Sox 9 (1:1 000, ab185230, Abcam, Cambridge, UK), Fgfr3 (1:100, F0425, Sigma), VEGF (1:200, ab46154, Abcam, Cambridge, UK), and PCNA (1:500, ab29, Abcam, Cambridge, UK), using the Dako Envision kit (Dako North America, Inc., Carpinteria, CA, USA).

For the mouse antibody against collagen X, pepsin was used for antigen retrieval (Sigma-Aldrich Co, St. Louis, MO, USA) for 2 h at 37 °C with post-natal and callus samples or for 1 h with embryo samples.

To detect DNA fragmentation, a TUNEL assay was performed on 6 µm sections after deparaffinisation using a Promega G3250 kit, according to the manufacturer’s protocol. Sections were mounted with Fluoromount-G mounting medium and 4’,6-diamidino-2-phenylindole (DAPI) for nuclear staining (00-4959-52, Life Technologies, Carlsbad, CA, USA).

To detect osteoclast activity, deparaffinized 6 µm sections were TRAP-stained using a Sigma 38717-1KT kit, according to the manufacturer’s protocol. Sections were counterstained with Aniline Blue, according to standard protocols.

Macroscopic analyses and linear measurements of the mandibles of mutants and control littermates were performed after Alizarin Red/Alcian Blue staining; according to standard protocols.

Images were captured with an Olympus PD70-IX2-UCB microscope (Olympus, Tokyo, Japan), and morphometric variables were analyzed with ImageJ software (National Institutes of Health, Bethesda, MD, USA).

### Spatial transcriptomic analyses

Using the non-stabilized mandibular fracture protocol described above, calluses from mutant mice and control littermates were analyzed on day 14 post-fracture. All samples were incubated in 4% paraformaldehyde for 4 h, fully decalcified in EDTA **(**pH 7.4) solution for 72 h and embedded in paraffin. Six µm serial sections were prepared, dried overnight at room temperature, and stored with desiccant at 4°C before further processing. Next, the sections were baked at 37°C overnight and then at 65°C for 3 h and then loaded onto a fully automated research stainer (Leica Bond RX) for further processing. The processing protocol included three main steps: slide baking, antigen retrieval for 20 min at 100°C, and treatment with Proteinase K (1.0 µg/mL in 1xPBS) for 15 min. A cocktail of probes (GeoMx Mouse Whole Transcriptome Atlas probes, targeting over 19 000 genes) was applied to each slide and allowed to hybridize at 37°C overnight in a humidity chamber. On the following day, slides were washed, blocked, and incubated with a combination of an Alexa Fluor 594 anti-alpha smooth muscle actin antibody (Abcam antibody ab202368; clone: 1A4) and Syto83 nucleic acid stain. Slides were stained for 1 h at room temperature in a humidity chamber, washed, and loaded onto the spatial multi-omic platform (GeoMx Digital Spatial Profiler).

### Next-generation sequencing and sequence data processing

The slides in the multi-omic platform were scanned for fluorescence, and ROIs were placed manually in the areas of newly formed bone and cartilage in the callus (Fig. 4a). Selection of cartilage ROIs was performed in the hypertrophic zone of the callus cartilage. Bone callus ROIs were selected in the trabecular newly formed bone, in proximity but not strictly adjacent to the callus cartilage. The ROIs were exposed to UV light (385 nm). The released indexing oligos were collected with a microcapillary and deposited on a 96-well plate. Samples were dried overnight and then resuspended in 10 µL of diethylpyrocarbonate-treated water. PCR was performed using 4 µL from each sample, and the oligos from each ROI were indexed using unique i5 and i7 dual-indexing systems (Illumina). The PCR reaction products were purified twice using AMPure XP beads (Beckman Colter, Inc.), according to the manufacturer’s instructions. Purified libraries were sequenced on an Illumina NovaSeq 6000 system. Fastq files were processed using the GeoMx NGS Pipeline (version 2.2, NanoString). Briefly, reads were trimmed to remove low-quality bases and adapter sequences. Paired end reads were aligned and stitched, and the barcode and unique molecular identifier (UMI) sequences were extracted. Barcodes were matched with known probe barcodes, with at most one mismatch allowed. Reads matching the same barcode were deduplicated by UMI. All analyses used UMI-deduplicated counts, to correct for any PCR amplification bias.

The spatial transcriptome assays were performed on two slides of 16 ROIs each, giving 32 ROIs in total. After the application of quality control filters, 27 sample ROIs from mandibular calluses were analyzed (Fig. 4a): 13 samples of cartilage (7 from mutants and 6 from controls) and 14 samples of newly formed bone (7 from mutants and 7 from controls). To increase the robustness of downstream statistical analyses, the three-step quality control filtering procedure selected ROIs with a sequencing saturation of more than 50 (to capture full sample diversity), removed outlier probes, and selected genes with an expression level above the limit of quantification (LOQ).

#### Data analysis and visualization

All statistical analyses and data visualizations were performed in an R software environment. Count data was processed and normalized using the GeoMxTools (v3.2), GeoMxWorkflows (v1.5,) and NanoStringQCPro (v1.30) R/Bioconductor packages. AOIs with fewer than 1 000 raw reads, a sequencing saturation <50%, an alignment rate <80% or a segment area <5 000 were filtered out of the analysis. For the negative control probes, outlier testing was performed and led to the removal of outlier probes from the analysis prior to collapsing counts. As described by Zimmerman et al., three criteria were used to determine whether a negative probe was an outlier^87^. Firstly, if the average count of a probe across all segments was less than 10% of the average count of all negative probes, then the probe was removed from all segments. Secondly, if the probe was considered to be an outlier in Grubb’s test (alpha = 0.01), it was removed locally (i.e. from that particular segment). Thirdly, if the probe was considered to be an outlier in Grubb’s test in over 20% of segments, it was removed from all segments. We calculated the geometric mean of the remaining probes, in order to collapse the negative probes into a single count value. The LOQ for a given gene was defined as two standard deviations above the geometric mean of negative probes. Only genes above the LOQ in at least 10% of ROIs were selected. Next, differential expression was analyzed using a combination of DESeq2^88^ and RUVSeq,^89^ after normalization against expression levels of mouse housekeeping genes. The obtained *P*-values were corrected for multiple testing by using the Benjamini-Hochberg procedure. A gene was considered to be significantly differentially expressed when the false discovery rate was below 10%. Lastly, pathway enrichment analysis was performed using GSEA^90^ and the Human Molecular Signatures database.

### In vivo preclinical experiments

Mutant mice received either BGJ398 (LC Laboratories (Wobum,MA USA); 4 mg/kg in DMSO 4% and isotonic saline serum), BMN111 (Biosynth, Louiseville, KY, USA; 0.8 mg/kg in isotonic saline serum) or vehicle by subcutaneous injection three times a week for 14 or 28 days after the fracture. The procedures for non-stabilized mandibular fractures, post-operative follow-up, morphometric analyses (CT scans and histomorphometry) and immunohistochemistry analyses were as described above.

### Statistical analysis

Statistical analyses were performed using GraphPad Prism software (version 8.00, GraphPad Software LLC, San Diego, CA, USA). The threshold for statistical significance was set to *P* < 0.05 (*0.01 < *P* < 0.05, **0.001 < *P* < 0.01, ***0.000 1 < *P* < 0.001, ****P < 0.000 1, in a Mann-Whitney test).

## Supplementary information

Supplemental material

## Data Availability

All data are available in the main text or the supplementary materials.
