# Supplementary material for: FGFR antagonists restore defective mandibular bone repair in a mouse model of osteochondrodysplasia
Source: Bone Res. 2025 Jan 21;13:12. doi: 10.1038/s41413-024-00385-x (PMC11751307; doi:10.1038/s41413-024-00385-x)
Supplement: Supplementary file 1 — Supplemental material [file 41413_2024_385_MOESM1_ESM.docx]

**FGFR antagonists restore defective mandibular bone repair in a mouse model of osteochondrodysplasia**

Anne Morice ^1^, Amélie de la Seiglière^1^, Alexia Kany^1^, Roman H Khonsari^1^, Morad Bensidhoum^2^, Maria-Emilia Puig-Lombardi ^3^, Laurence Legeai Mallet^1*^.

1. Université de Paris Cité, Imagine Institute, Laboratory of Molecular and Physiopathological Bases of Osteochondrodysplasia, INSERM UMR 1163, Paris, France
2. B3OA UMR CNRS 7052, Université Paris Cité, Paris, France.
3. Bioinformatics Core Platform, Imagine Institute, INSERM UMR1163 and Structure Fédérative de Recherche Necker, INSERM US24/CNRS UAR3633, Université Paris Cité, Paris, France

**Conflict of interest statement**

The authors have declared that no conflict of interest exists.

**Supplemental Materials**

**Fig. S1**


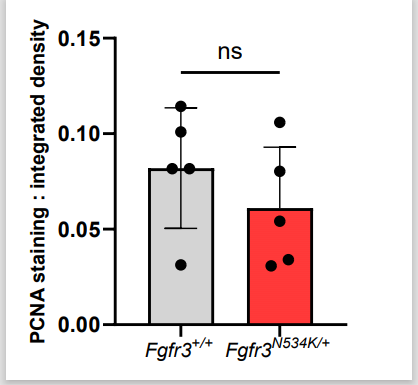


**Fig. S1:** Meckel’s cartilage at E13.5: graphical representation of the PCNA staining density in MC from *Fgfr3^+/+^* (n=5) and *Fgfr3^N534K/+^* mice (n=5). ns: non-significant.

**Fig. S2**


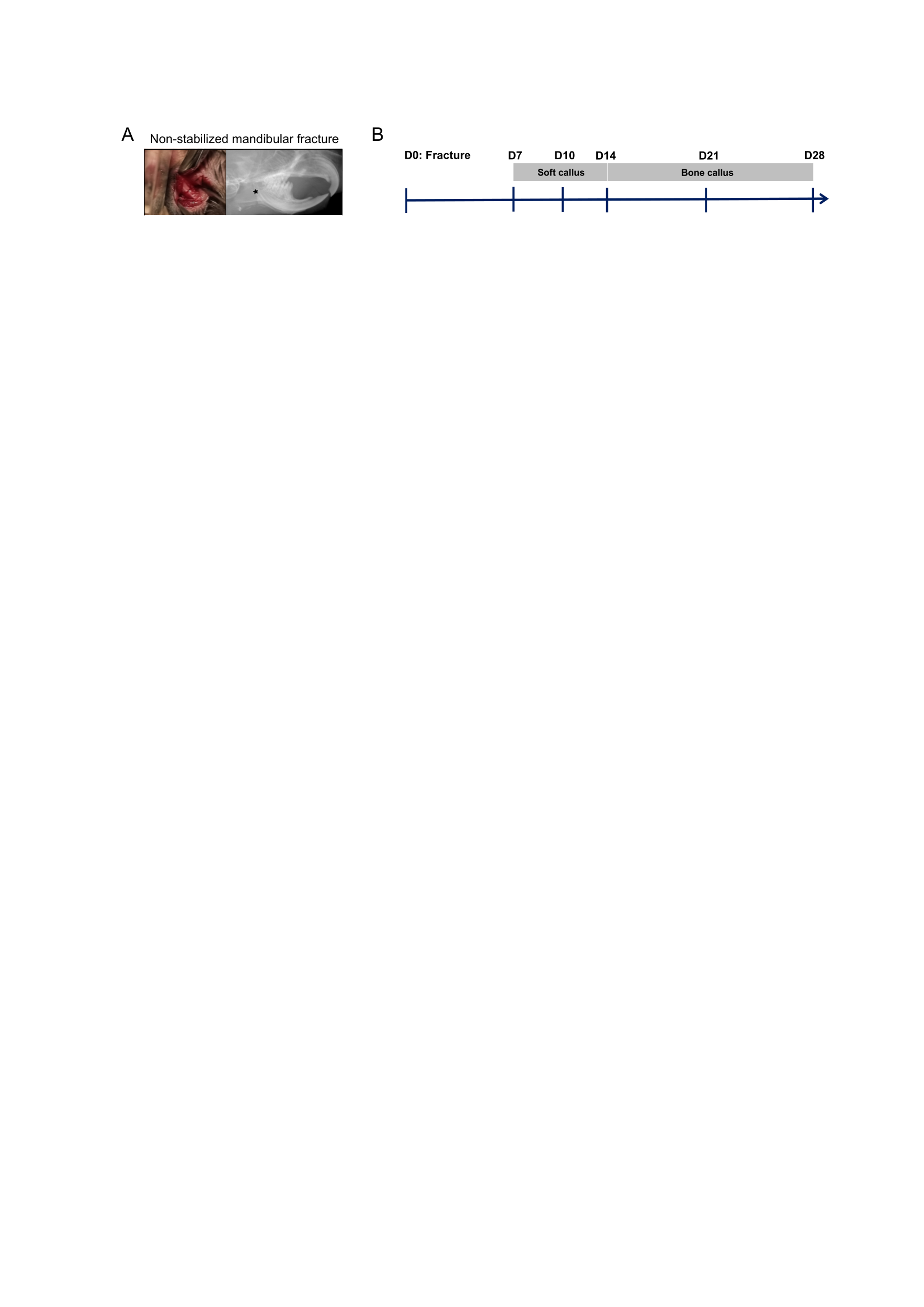


**Fig. S2:** A – Images of the submandibular cutaneous approach to the mandibular ascending branch. X-rays of the skull of a P42 *Fgfr3^+/+^* mouse (sagittal view), showing the direction of the non-stabilized mandibular fracture from the basilar border to the coronoid notch (
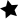
). B – Schematic representation of bone repair analysis after fracture on day (D)0, with the key points for endochondral bone repair on D7 and D10) (soft callus), D14 (a transition between a soft callus and a bone callus), D21, and D28 (bone callus).

**Fig. S3**

**
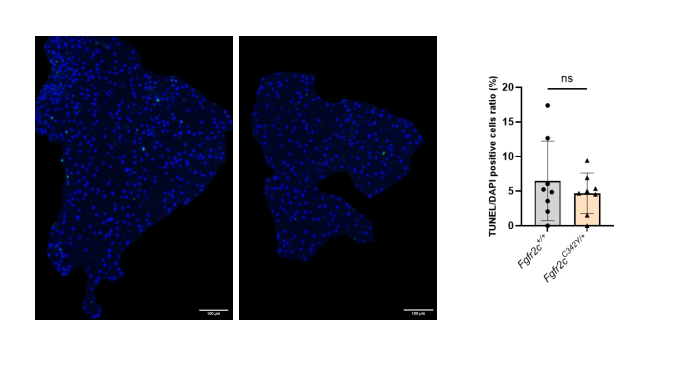
**

**Fig. S3:** Representative images of TUNEL/DAPI staining (green fluorescence) of hypertrophic cartilage area (containing collagen-X-positive chondrocytes) in samples from *Fgfr2^+/+^* and *Fgfr2c^C342Y/+^* mice, on D14 post-fracture. A graphical representation (in %) of the numbers of TUNEL- and DAPI-positive cells in the callus of *Fgfr2^+/+^* (n=7) and *Fgfr2c^C342Y/+^* mice (n=8) (scale bar: 100 µm).

**Fig. S4:**

**Fig. S4:** A. Representative PCNA labelling of callus cartilage on day 7 post-fracture in *Fgfr3^+/+^* and *Fgfr3^N534K/+^* mice (scale bar: 100 µm). A graphical representation of PCNA quantification. ns: non-significant.

B. Representative images of callus cartilage labelled for collagen type II and VEGF on day 14 post-fracture in *Fgfr3^+/+^* and *Fgfr3^N534K/+^* mice (scale bar: 200 µm).

C. Representative Sox9, Fgfr3 and VEGF labelling of callus cartilage on day 14 post-fracture in *Fgfr3^+/+^* and *Fgfr3^N534K/+^* mice (treated with vehicle, BMN111, or BGJ398; scale bar: 200 µm).

**Fig. S5**

**Fig. S5:** Gene set enrichment analysis based on the functional annotation (Gene Ontology – Biological Process pathways) of genes deregulated in samples of (A) cartilage and (C) bone from *Fgfr3^N534K/+^* mice. The size of the dot corresponds to the number of significantly deregulated genes (left panel: upregulation; right panel: downregulation) in a given pathway. The color scale indicates the significance of the enrichment (adjusted *P*-values from hypergeometric tests). Violin plots show the expression levels (as normalized read counts) of specific cartilage markers (B: *Col2a1, Col10a1, Ihh (*coding for Indian Hedgehog*), Sox9, Mapk3, Pth* (coding for parathormone), and *Bglap*) and bone markers (D: *Col1a1, Sp7 (*coding for Osx*), Runx2, Alpl* (coding for alkaline phosphatase)*, Spp1* (coding for osteopontin), and *Tnfrsf11a* (coding for Rank) in *Fgfr3^N534K/+^* and *Fgfr3^+/+^* mice.

**Fig. S6**


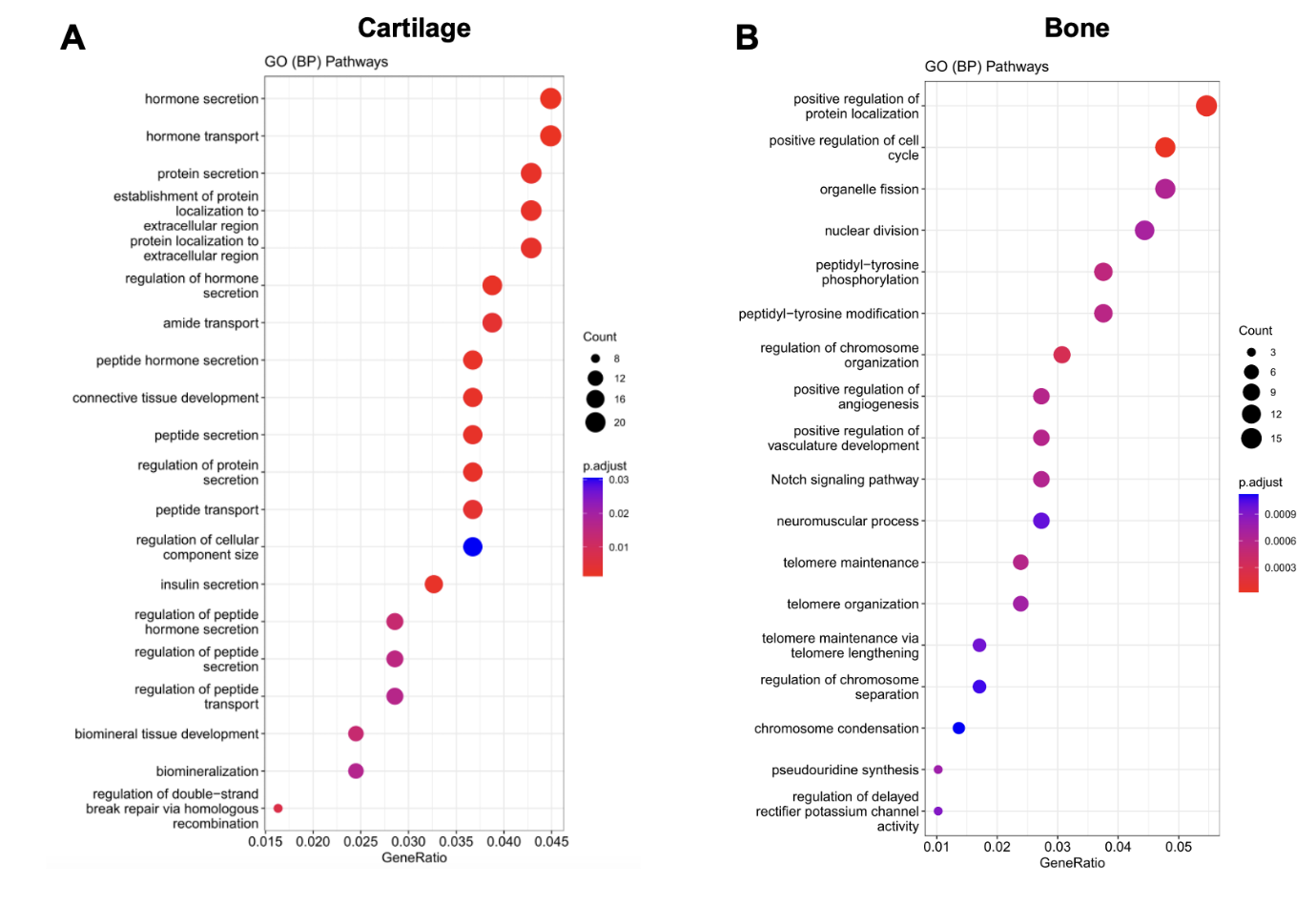


**Fig. S6:** Gene Ontology – Biological Process Pathway (GO, BP) Enrichment Analysis. Each dotplot displays the Gene Ontology (GO, BP – Biological Process term database) pathway enrichment analysis results for genes significantly upregulated in**A**, cartilage tissue or **B**, bone tissue. The x-axis shows the gene ratio, which is the proportion of upregulated genes involved in each term relative to the total number of upregulated genes in the analysis. The y-axis lists the GO terms. Dot size reflects the number of upregulated genes associated with the GO term, and dot color indicates the adjusted p-value, with a color gradient from red (highest significance) to blue (lowest significance).

**Figure S7**

**
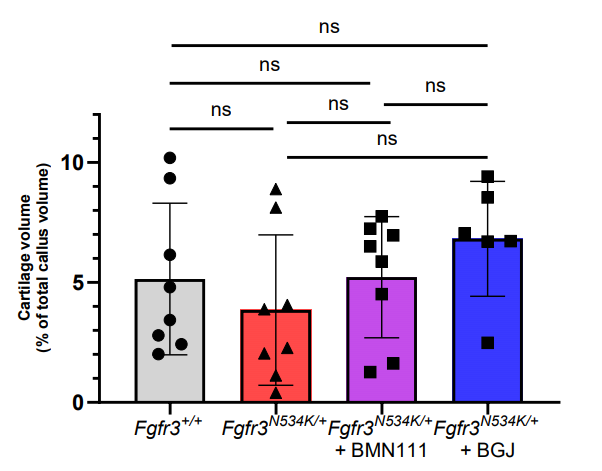
**

**Fig. S7:** A graphical representation of histomorphometric cartilage volume data on D14 post-fracture, based on Alcian Blue/Sirus Red staining (n=8 for *Fgfr3^+/+^*, n=8 for *Fgfr3^N534K/+^*, n=8 for *Fgfr3^N534K/+^* + BMN111, n=6 for *Fgfr3^N534K/+^* + BGJ398).

**Fig. S8**

**
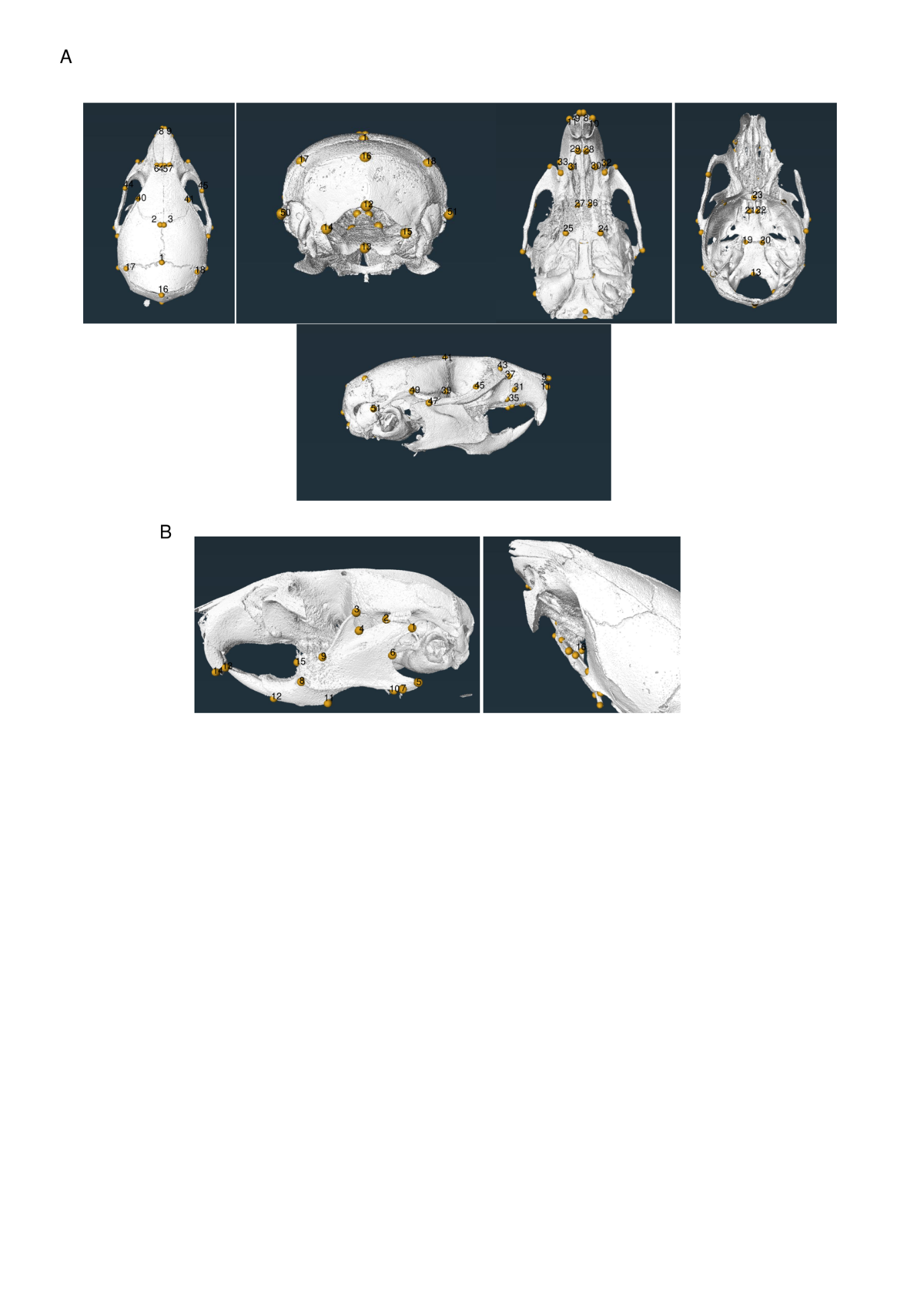
**

**Fig. S8:** Reconstructed 3D µCT images of the skull (A) and the mandible (B) of a wild-type mouse on P42, showing the positions of skull and mandibular landmarks used in the morphometric analyses.

**Supplemental File 1:** Definitions of skull and mandibular landmarks.

**Anatomical skull and mandibular landmarks: definition**

**Skull landmarks**

1. Most anterior point on the ectocranial surface of the interparietal on the sagittal plane
2. Most medio-anterior point of the parietal (L)
3. Most medio-anterior point of the parietal (R)
4. Most postero-medial point of the nasal bone (L)
5. Most postero-medial point of the nasal bone (R)
6. Most postero-lateral point of the nasal bone (L)
7. Most postero-lateral point of the nasal bone (R)
8. Most antero-medial point of the nasal bone (L)
9. Most antero-medial point of the nasal bone (R)
10. Most supero-anterior point of the maxilla accounting for the lateral part of the nasal aperture (L)
11. Most supero-anterior point of the maxilla accounting for the lateral part of the nasal aperture (R)
12. Mid-point on the posterior margin of the foramen magnum, taken on squamous occipital
13. Mid-point on the anterior margin of the foramen magnum, taken on basioccipital (basisphenoid)
14. Most infero-lateral point on the squamous occipital (L)
15. Most infero-lateral point on the squamous occipital (R)
16. Most posterior point (midline) on the ectocranial surface of the interparietal
17. Most lateral point of the ectocranial surface of the interparietal (left)
18. Most lateral point of the ectocranial surface of the interparietal (right)
19. Most antero-lateral point on the corner of the basioccipital (L)
20. Most antero-lateral point on the corner of the basioccipital (R)
21. Most anterolateral point on the sphenoid (L)
22. Most anterolateral point on the sphenoid (R)
23. Most antero-medial point on the body of the presphenoid
24. Most lateral point of the junction between the posterior edge of the palatine plate and the pterygoid plate (L)
25. Most lateral point of the junction between the posterior edge of the palatine plate and the pterygoid plate (R)
26. Most posterior point of the anterior palatine foramen (L)
27. Most posterior point of the anterior palatine foramen (R)
28. Most anterior point of the anterior palatine foramen (L)
29. Most anterior point of the anterior palatine foramen (R)
30. Most infero-medial point of the premaxillary-maxillary suture, taken on premaxilla (L)
31. Most infero-medial point of the premaxillary-maxillary suture, taken on premaxilla (R)
32. Most lateral point on the margin of the premaxillary-maxillary suture, taken on premaxilla, at the midline (L)
33. Most lateral point on the margin of the premaxillary-maxillary suture, taken on premaxilla, at the midline (R)
34. Most inferior point of the infraorbital hiatus (L)
35. Most inferior point of the infraorbital hiatus (R)
36. Most distal point of the infraorbital hiatus (L)
37. Most distal point of the infraorbital hiatus (R)
38. Most superior point on the squamous temporal, intersection of the coronal suture (L)
39. Most superior point on the squamous temporal, intersection of the coronal suture (R)
40. Most lateral intersection of the frontal and parietal, taken on the frontal (L)
41. Most lateral intersection of the frontal and parietal, taken on the frontal (R)
42. Most superior point of the infraorbital hiatus (L)
43. Most superior point of the infraorbital hiatus (R)
44. Intersection of zygoma with zygomatic process of maxilla, taken on zygoma (L)
45. Intersection of zygoma with zygomatic process of maxilla, taken on zygoma (R)
46. Intersection of zygoma with zygomatic process of temporal, taken on zygoma (L)
47. Intersection of zygoma with zygomatic process of temporal, taken on zygoma (R)
48. Basis of the zygomatic process of the temporal (L)
49. Basis of the zygomatic process of the temporal (R)
50. Most posterior point on the posterior extension of the forming squamosal (L)
51. Most posterior point on the posterior extension of the forming squamosal (R)

**Mandibular landmarks**

1 Posterior tip of the mandibular condyle

2 Anterior tip of the mandibular condyle

3 Tip of the coronoid process

4 Greater concavity of the sigmoid notch

5 Tip of the mandibular angle

6 Greater concavity of the ramus

7 Masseter posterior insertion

8 Masseter anterior insertion

9 Anterior tip of the external oblique line

10 Most inferior point of the basilar contour (posterior)

11 Most inferior point of the basilar contour (anterior)

12 Most posterior point of the mandibular incisor on the inferior side

13 Tip of the mandibular incisor

14 Tip of the maxillary incisor

15 Most anterior point of the first mandibular molar

16 Most posterior point of the third mandibular molar

17 🡺 29: Contralateral mandibular landmarks
